# Inequalities in the coverage of place of delivery and skilled birth attendance: analyses of cross-sectional surveys in 80 low and middle-income countries

**DOI:** 10.1186/s12978-016-0192-2

**Published:** 2016-06-17

**Authors:** Gary Joseph, Inácio Crochemore Mohnsam da Silva, Fernando C. Wehrmeister, Aluísio J. D. Barros, Cesar G. Victora

**Affiliations:** International Center for Equity in Health, Post-Graduate Program in Epidemiology, Federal University of Pelotas, Rua Marechal Deodoro, 1160, 3o andar, Pelotas, RS 96020-220 Brazil

**Keywords:** Skilled delivery, Maternal health services, Skilled birth attendance, Birth attendance, Delivery assistance, Low and middle-income countries, Developing countries, Global health

## Abstract

**Background:**

Having a health worker with midwifery skills present at delivery is one of the key interventions to reduce maternal and newborn mortality. We sought to estimate the frequencies of (a) skilled birth attendant coverage, (b) institutional delivery, and (c) the combination of place of delivery and type of attendant, in LMICs.

**Methods:**

National surveys (DHS and MICS) performed in 80 LMICs since 2005 were analyzed to estimate these four categories of delivery care. Results were stratified by wealth quintile based on asset indices, and by urban/rural residence. The combination of place of delivery and type of attendant were also calculated for seven world regions.

**Results:**

The proportion of institutional SBA deliveries was above 90 % in 25 of the 80 countries, and below 40 % in 11 countries. A strong positive correlation between SBA and institutional delivery coverage (rho: 0.97, *p* <0,001) was observed. Eight countries had over 10 % of home SBA deliveries, and two countries had over 10 % of institutional non-SBA deliveries. Except for South Asia, all regions had over 80 % of urban deliveries in the institutional SBA category, but in rural areas, only two regions (CEE & CIS, Middle East & North Africa) presented average coverage above 80 %. In all regions, institutional SBA deliveries were over 80 % in the richest quintile. Home SBA deliveries were more common in rural than in urban areas, and in the poorest quintiles in all regions. Facility non-SBA deliveries also tended to be more common in rural areas and among the poorest.

**Conclusion:**

Four different categories of delivery assistance were identified worldwide. Pro-urban and pro-rich inequalities were observed for coverage of institutional SBA deliveries.

**Electronic supplementary material:**

The online version of this article (doi:10.1186/s12978-016-0192-2) contains supplementary material, which is available to authorized users.

## Background

Globally, it is estimated between 1990 and 2015, 10.7 million women died from complications related to pregnancy and childbirth [1]. Besides, 2.8 million newborns died annually within 28 days of birth, with 2 million occurring within the first week of life [2, 3], and there are 2.6 million stillbirths of which 45 % occur during childbirth or labor [4]. Most of these deaths (99 %) and complications occur in LMICs, due to causes that are usually preventable [5]. However, laudable progress have been observed and the global maternal mortality rate (MMR) fell from 385 deaths per 100 000 livebirths in 1990 to 216 in 2015, corresponding to a relative decline of 43.9 % [1]. The neonatal mortality rate fell from 33 deaths per 1000 live births in 1990 to 20 in 2013, and stillbirths rate from 24.7 per 1000 live births in 2000 to 18.4 in 2015 [6]. Progress also have been observed in the countdown countries with reduction of maternal mortality ratio of 45 % over the past two decades [7].

To reduce these maternal, fetal and newborn deaths, several initiatives have been launched [8]; these include ensuring that deliveries are assisted by SBAs, and the extension of institutional deliveries coverage [9].

In 2000, maternal health was included as one of the Millennium Development Goal (MDG5), with the target of reducing the maternal mortality rate by three quarters by 2015 [8]. The proportion of births attended by a SBA was considered as a key indicator for monitoring progress on maternal and newborn health [10]. However, the MDGs have been criticized due to lack of emphasis on inequalities and recent efforts are underway to measure progress towards universal health care from an equity perspective [11, 12].

At a special session held by the United Nations General Assembly, it was agreed that all countries should increase their efforts to reach 80 % (by 2005) and 90 % (by 2015) coverage of skilled birth attendance [13]. Since then, countries have employed several strategies to achieve these goals [14]. However, there is variation among countries about the proper definition of SBA and the most appropriate place for delivery assistance. Some countries invested in training health care professionals to increase the coverage of institutional deliveries or home deliveries by a SBA. Other countries invested in providing some formal training to traditional birth attendants (TBAs) such as matrones [15].

The first attempt at defining SBA was made by the WHO/UNFPA/UNICEF/World Bank in 1999. However, it has been criticized for failing to refer to the place of delivery and therefore being biased towards facility births [16]. In 2004, a joint statement by WHO, the International Confederation of Midwives (ICM) and the International Federation of Gynecology and obstetrics (FIGO), proposed a more refined definition of a SBA relative to what had been first proposed in 1999 [17]. SBAs were defined as accredited health professionals such as midwifes, doctors or nurses who have been educated and trained to proficiency in the skills needed to manage normal (uncomplicated) pregnancies, childbirth and the immediate postnatal period, and in the identification, management and referral of complications in women and newborns [17]. It was also recommended that deliveries should take place in a range of appropriate settings, from home to tertiary referral centre, depending on availability and need [18].

The term TBA (also known as traditional midwife, community midwife, matrones or rural auxiliary midwife, among others) refers to a health care provider who usually has not received any formal training, and who does not have professional certification or licensure [19, 20]. TBAs often work in rural and remote areas. Because of their access to such underserved communities, some countries, training institutions and non-governmental organizations have initiated efforts to TBAs in basic and emergency obstetric and other maternal health topics in order to strengthen the links between health services and communities, and thus improve health outcomes among women and newborns [15, 21]. Several countries regard TBAs who received some amount of formal training as SBAs, and include them in the primary health system [22]. The variability in the definition of SBAs has to be kept in mind when carrying out international comparisons [22, 23].

The recommendation on place of delivery, however, is controversial [24, 25]. Whereas some suggest that home delivery should be encouraged for women with low-risk, under the supervision of a SBA, others argue that pregnancy is always associated with risk and recommend institutional delivery for all. Yet, a third group argues that the place of delivery should be decided upon as a joint judgment between patients and professionals.

Monitoring of global SBA coverage showed an increase from 61.5 % in 2000 to 73 % in 2013 [26]. However, important inequalities remain between and within-country, with SBA coverage being the least equitable indicator related to maternal and newborn health [27, 28]. Developed countries had over 99 % coverage on SBA, while South Asia and the Sub-Saharan Africa had only 53 and 51 % coverage respectively [26]. While some countries have reached coverage over 90 % since 1990, many others are still struggling, even at the national aggregate level [29]. Place of residence (urban/rural) and household wealth are two keys dimension of inequalities in SBA coverage [30]. According to Channon et al., countries that have achieved high coverage of maternal health care by SBA from a relatively low baseline over the last decades have progressed through a common pathway. Further, the coverage has increased first among the urban rich, followed by the rural rich, the urban poor and the rural poor the last to be reached [30].

A review of the literature and preliminary analyses from various countries led to the identification of four categories of delivery assistance: 1) institutional delivery by a SBA; 2) home births by a SBA; 3) institutional delivery by an unskilled health provider [31, 32]; and 4) home births by an unskilled health provider [33, 34]. The present analyses were aimed at describing between and within-country inequalities in (a) skilled birth attendant coverage, (b) institutional delivery, and (c) the combination of place of delivery and type of attendant in LMICs.

## Methods

### Design and data sources

This study was based on publicly available data sets from large cross-sectional surveys nationally representative including Demographic Health Surveys (DHS) and the Multiple Indicator Cluster Surveys (MICS). Both types of surveys use standardized questionnaires, to collect information from women on birth attendance, place of delivery, childbirth, as well as on individual, household and community characteristics that allow for comparison of the result across countries. The surveys are typically conducted and implemented by the national central statistic agencies. Each survey contains information provided by women of reproductive age from 15 to 49 years old. Ethical approval was the responsibility of the institution that commissioned, funded, or carried out the surveys, which ensured the complete confidentiality of respondents. Details on DHS and MICS are available elsewhere [35, 36].

We used the latest available surveys from 80 LMICs belonging to the seven world regions as defined by the United Nations Children’s Fund (UNICEF) [36], and for which information were available for birth attendant and place of delivery as of 2005. Of these countries, 55 had DHS and 25 had MICS. For MICS, data were obtained from the child file and participants were women aged from 15 to 49 years with at least one live birth in the last two years. For DHS, data were obtained from the woman’s file and participants were women aged from 15 to 49 years with at least one live birth in the last 3 years; such limits of time periods were intended to avoid recall bias, and it is practically the only difference in terms of data collection on this topic. For both surveys, these files were matched with the household files that include the asset indices.

### Outcomes

Two outcomes relative to childbirth were analyzed: delivery by a SBA and the place of delivery.

SBAs include doctors, nurses, midwifes and other cadres that individual countries recognize as such (auxiliary midwife, auxiliary nurse, community health officer) [13, 22]. Information on birth attendance in the survey questionnaires were collected through unprompted answers to the question “*Who assisted with the delivery of (NAME OF THE CHILD)?”.* Examples of the actual questions used in DHS and MICS are included in Additional file 1: Appendix A.

Regarding place of the delivery in both DHS and MICS questionnaires, the discrete nominal response variable was as followed: home (respondent’s home or another non-institutional setting); Public sector (government hospital, government health centre, government health post or other public sector); or private medical sector (private hospital or clinic, other private medical facility). Both public and private sector deliveries were considered as “institutional”. These information were obtained from a face-to-face application questionnaire with open response option (i.e. allowed multiple provider to be indicated per delivery) [35, 36].

These two outcome variables were then combined and categorized into four categories of delivery assistance: 1) institutional, SBA; 2) institutional, non-SBA; 3) home, SBA; 4) and home, non-SBA. These news variables are referred to as *“combination of place of delivery and type of attendant.”*

Another important category of delivery that has recently become a focus of interest in some countries is the women who deliver absolutely alone with “no one present (NOP)” [37]. In our analyses, we opted not to include this category because data on NOP were not available for most countries.

### Stratification variables

Two main stratifiers were considered in this study: place of residence of the women and the wealth index scores. Place of residence was coded as either urban or rural. As direct measures of living standards such as income, expenditure and consumption are rarely collected in the DHS and MICS surveys. These surveys collect information on household assets and characteristics of the dwelling, that can be used as a proxy measure for living standards, known as asset or wealth index [38, 39]. We used the wealth index scores based on Principal Component Analyses, calculated by the original DHS and MICS survey team for each household and presented in quintiles [39]. Quintile 1 (Q1) represents the poorest 20 % of households in the survey sample and quintile 5 (Q5) represents the richest 20 %.

### Statistical analysis

Descriptive analyses were carried out for each country to estimate the frequencies of (a) SBA coverage, (b) institutional delivery, and (c) the combination of place of delivery and type of attendant. Analyses were stratified according to the seven UNICEF world regions. Pearson’s correlation was used to calculate the association between SBA and institutional deliveries at country level. Significance testing of the association between the outcomes and place of residence and wealth quintiles, at individual level, was done using chi-squared tests for heterogeneity and for linear trends for a subset of countries with unusual pattern of delivery assistance (see below). When a proportion was equal to zero or 100 %, exact binomial confidence intervals were calculated.

All analyses were carried out with STATA (version 13.1) and EXCEL 2013, taking into account the sampling design characteristics of each survey and the sample weights. When calculating regional mean values, we opted not to use country weights because information was missing for several countries in some regions.

## Results

In the 80 countries studied, 73.8 % (±23.2 SD) of births were assisted by SBAs, and 70.5 % (±24.6 SD) were inside a health institution.

**Fig. 1:**
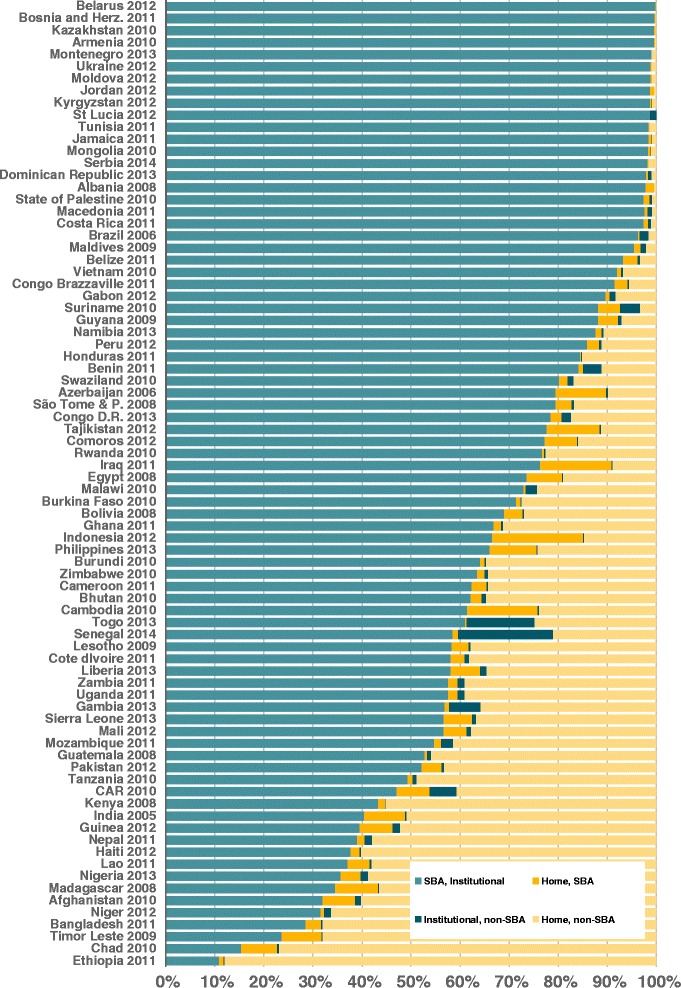
Combination of skilled birth attendance (SBA) and institutional deliveries in low and middle-income countries in the most recent survey, by country

Figure 1 shows the description of the four categories of delivery assistance in the most recent surveys by country. For 25 out of the 80 countries, the proportion of deliveries by a SBA in a health facility was above 90.0 %. However, 11 countries have fewer than 40.0 % of births in this category. All of the latter are low-income countries according to the World Bank classification.

Six countries - Azerbaijan (10.3 %), Cambodia (14.4 %), Indonesia (18.5 %), Iraq (14.5 %), Philippines (10.0 %) and Tajikistan (10.8 %) - had more than 10 % of all births assisted by a SBA outside a health facility. Two countries - Senegal (19.4 %) and Togo (13.8 %) - had over 10.0 % of all births carried out in a health facility by a non-SBA.

**Fig. 2:**
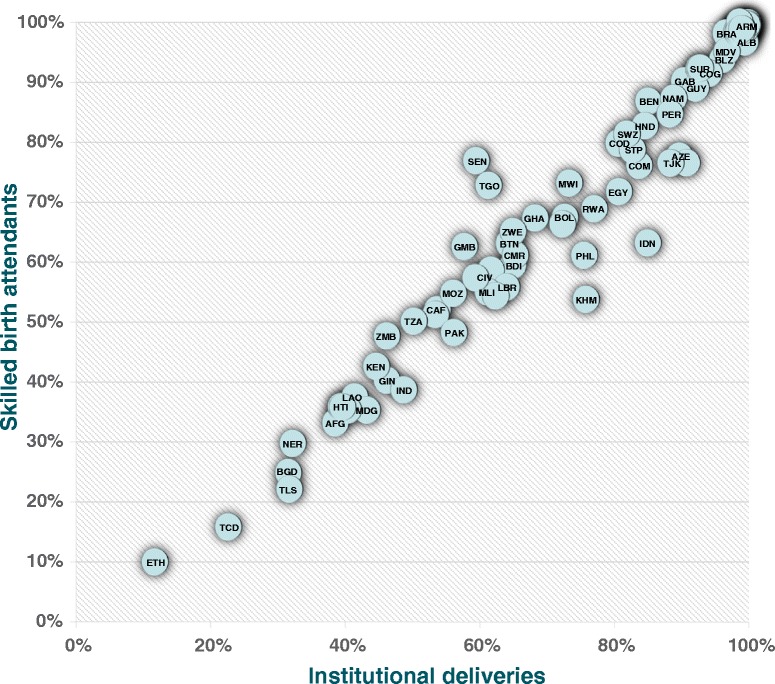
Association between SBA and institutional delivery coverage in low and middle-income countries (see Additional file 5 for country codes) (Pearson correlation coefficient = 0.97, *P* <0.001)

Figure 2 shows a strong positive correlation between SBA and institutional delivery coverage, by country (Pearson’s correlation: 0.97, *p* <0,001). In the 80 countries studied, 98.3 % of institutional deliveries are performed by a SBA, and 95.8 % of SBA deliveries are in an institution. Eight countries (Azerbaijan, Cambodia, Indonesia, Iraq, Philippines, Tajikistan, Senegal and Togo) are outliers, and will be discussed below.

Descriptions of the four categories of delivery assistance according to place of residence and wealth quintiles with the corresponding significance levels are presented in appendix (Additional file 2: web appendix tables A1 and A2).

**Fig. 3:**
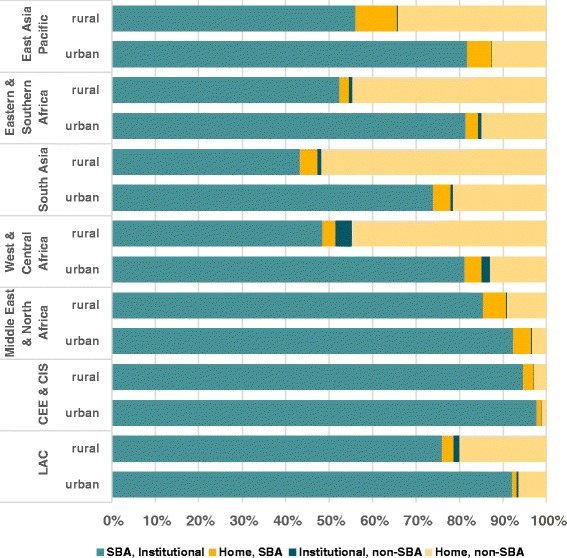
Combination of place of delivery and type of professional, by urban/rural residence, average values according to UNICEF regions (unweighted average of country results)

Figure 3 shows the unweighted average value of the four categories of delivery assistance by world region, according to place of residence (urban or rural). Except for South Asia, all regions have over 80.0 % of urban deliveries by a SBA in a health facility. In rural areas, only two regions (CEE & CIS, Middle East & North Africa) present average coverage above 80.0 %. Home SBA deliveries are more common in rural than in urban areas in all regions. However, regional averages may hide important differences among countries (see Additional file 2: web appendix figures A1–A7). For example, home SBA deliveries are common in the rural areas of countries such as Peru (5.6 %), Suriname (6.1 %) and Bolivia (7.4 %) (Additional file 2: Figure A1); Philippines (10.0 %), Cambodia (15.6 %) and Indonesia (26.5 %) (Additional file 2: FigureA2); and in Azerbaijan (15.6 %) and Tajikistan (12.2 %) (Additional file 2: Figure A3). In contrast, home SBA deliveries are common in the urban areas of Afghanistan (9.4 %), Guinea (13.6 %), Chad (15.8 %) and Madagascar (21.2 %) (Additional file 2: Figure A4–A6). In Egypt and Iraq, home SBA deliveries were common in both urban and rural areas (Additional file 2: Figure A7).

**Fig. 4:**
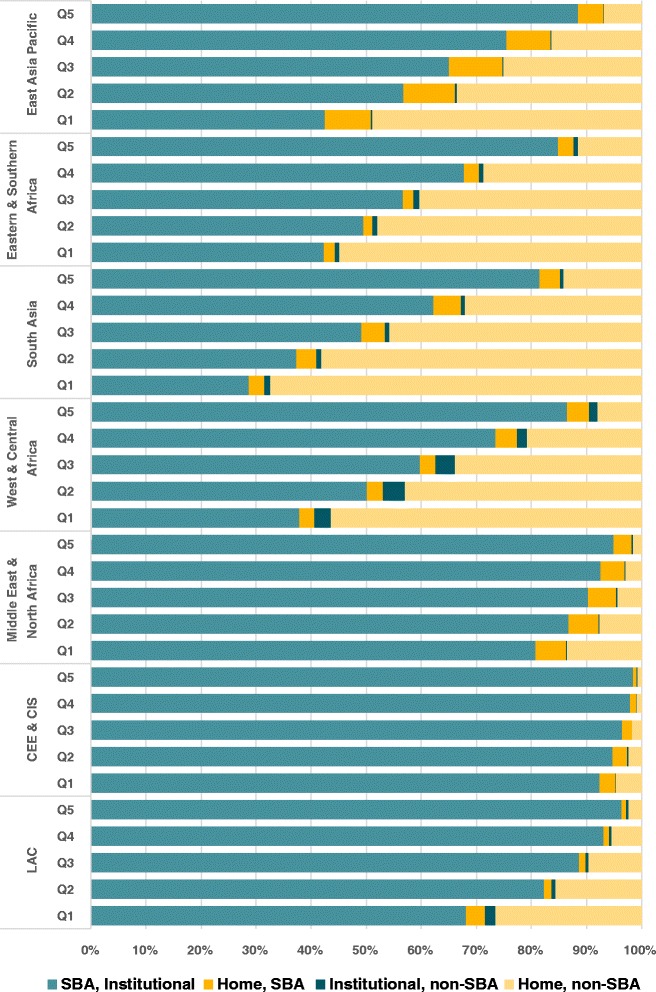
Combination of place of delivery and type of professional, by wealth quintile, average values according to UNICEF region (unweighted average of country results)

Results stratified by wealth quintiles are shown in Fig. 4. In all regions, births in a health facility by a SBA were more common in the richest quintiles, while home deliveries (with or without a SBA) were more common in the poorest quintiles. Despite a relative lower prevalence, facility deliveries by unskilled health workers also tended to be more common among the poor. In East Asia Pacific, 8.3 % of deliveries in the poorest quintile are home SBA, but in other regions these represent only 3.0 %. Important differences were also observed among countries: Indonesia and Tajikistan have more than 15.0 % of home SBA deliveries in the poorest quintiles, while in Madagascar the situation is reversed, with 23.6 % in the richest quintile (Additional file 2: web appendix figures B1-B7).

**Fig. 5:**
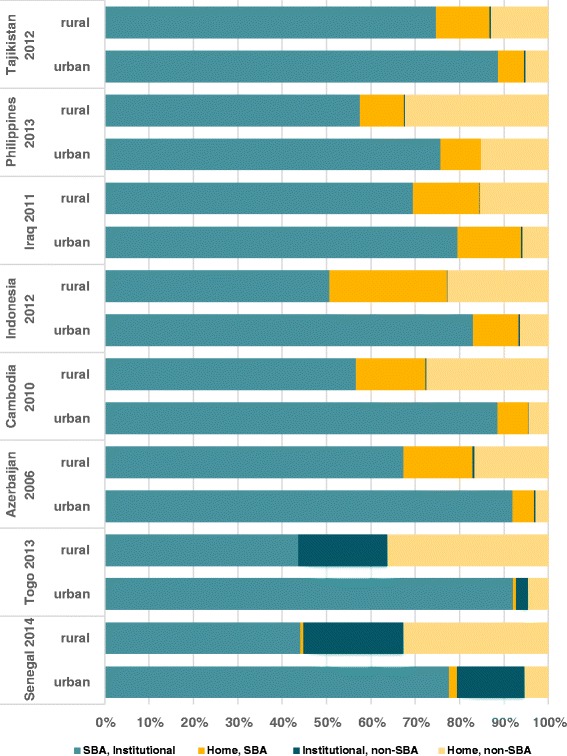
Distribution of place of delivery and type of professional in countries with at least 10 % of home SBA deliveries (Azerbaijan, Cambodia, Indonesia, Iraq, Philippines and Tajikistan) or at least 10 % of institutional deliveries by an unskilled worker (Senegal and Togo), by place of residence

**Fig. 6:**
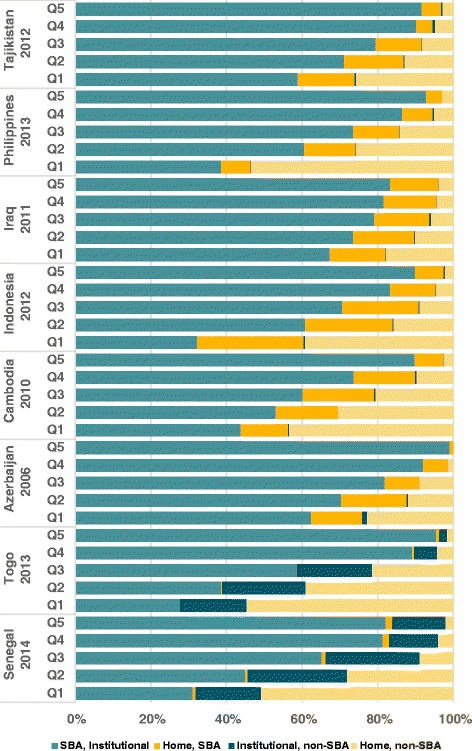
Distribution of place of delivery and type of professional in countries with at least 10 % of home SBA deliveries (Azerbaijan, Cambodia, Indonesia, Iraq, Philippines and Tajikistan) or at least 10 % of institutional deliveries by an unskilled worker (Senegal and Togo), by wealth quintile

Countries with more than 10.0 % of either home SBA delivery (Tajikistan, Philippines, Iraq, Indonesia, Cambodia and Azerbaijan), or facility non-SBA deliveries (Togo and Senegal) were analyzed separately (Figs. 5 and 6). Home SBA deliveries were more pronounced in rural areas. In Senegal, facility non-SBA deliveries were common in both urban and rural areas (15.2 and 22.6 % respectively); while in Togo, these were more frequent in rural areas (20.1 %) (Fig. 5). All differences were significant except for Iraq (*p* = 0,93) and Philippines (*p* = 0,23) (Additional file 3: Web appendix C).

Figure 6 shows a similar analysis by wealth quintiles. In the poorest quintiles, home SBA deliveries vary from 12.6 % in Cambodia to 28.1 % in Indonesia, and in the richest quintiles, this proportion goes from 0.9 % in Azerbaijan to 12.6 % in Iraq. In both Senegal and Togo, facility births by a non-SBA were more common in the three poorest quintiles.

Pro-poor patterns were observed for home SBA and institutional deliveries by an unskilled worker, except for Iraq (*p*: 0.071) (Additional file 4: Web appendix D).

## Discussion

Place of delivery and type of attendant are both important determinants of maternal and newborn health [7, 26]. The purpose of this study was to estimate the frequencies of (a) SBA coverage, (b) institutional delivery, and (c) the combination of place of delivery and type of attendant in LMICs. Similar analyses were recently published for 57 countries using DHS data, with a focus on the private and public sectors in four world regions [40]. We present analyses for 80 countries with data from both MICS and DHS, stratified by place of residence and wealth asset index. Our results are analyzed according to seven world regions using the UNICEF classification. Place of delivery and type of attendant were combined into four categories of delivery assistance, to allow for the possibilities that deliveries in health facilities are not necessarily performed by SBAs, and that deliveries outside a health facility do not necessarily represent unskilled birth attendance. We did not find any systematic studies on these four categories of delivery assistance in the literature.

We show that coverage of SBA at delivery varies widely across LMICs. In 25 out of the 80 countries studied, the national coverage was over 90.0 %; these countries have reached the 2015 target proposed by the United Nations General assembly in 1999 [22, 41]. On the other extreme, 11 low-income countries [42] have national coverage below 40 %. These results are consistent with recent findings in the literature [29, 40, 42, 43]. In addition, pro-urban and pro-rich inequalities were documented, both at the national and regional level. These results are also consistent with other recent publications [29, 44, 45].

When we analyzed the correlations between SBA coverage and institutional deliveries at country level, two groups of countries were outliers. Azerbaijan, Cambodia, Indonesia, Philippines, Iraq and Tajikistan the prevalence of home SBA deliveries was over 10.0 %, being higher in rural areas and in the poorest quintiles. In Senegal and Togo, the proportion of births in a health facility by unskilled birth attendants was above 10.0 %, being higher in the poorest quintiles and – in Togo – in rural areas. We sought to understand why these categories are so prevalent in these two groups of countries.

Since the launch of the Safe Motherhood Initiative in 1987 and of the Millennium Development Goals in 2000, countries have adopted different strategies to improve maternal and newborn health outcomes [10, 46, 47]. In several countries, delivery by SBAs outside health facilities have been promoted. In the Philippines, “*birthing homes*” supervised by public or private health facilities provide birthing services including antenatal, normal spontaneous delivery, and postnatal care, particularly in rural and poor population [44, 45]. This service is provided by accredited health personnel, generally midwives, with a minimum of 2 years training [48–50]. In Indonesia, the situation is very similar except that the duration of the training program is one year [51, 52]. In Cambodia, no formal program about home SBA is reported in the literature. According to Por et al., home SBA deliveries are driven by economic interest from government health personnel, who can charge women for the delivery at home, but not in a public facility [53, 54]. In Tajikistan, health care has deteriorated dramatically since 1991 [55, 56], and most rural maternities are operating without the basic conditions such as heat or running water. Thus, women consider giving birth at home safer, more comfortable and affordable than in a facility [55–57]. In contrast, in Azerbaijan, a system known as “*feldsher-accoucher* points” staffed by mid-level health care providers who focus on primary health care in rural areas is in charge of assisting deliveries at home [57, 58]. In Iraq, due to decades of war and economic sanctions, many health facilities have faced serious difficulties in keeping functioning and providing adequate health care [59]. Besides, the fear of terrorist attacks has reportedly led patients to avoid public spaces such as health facilities, and some women feel more secure by requesting a health care provider to assist their births at home [59]. These are likely the reasons why home SBA deliveries are so prevalent in these countries, especially in rural areas and in the poorest quintiles.

A different pattern was observed in West-African countries such as Senegal and Togo, where more than 10 % of institutional deliveries were carried out by unskilled birth attendants. According to Kodio et al., in some villages in Senegal, no qualified midwife or nurse are presented in the health facility and deliveries are generally assisted by a traditional birth attendant (known as “matrone” in French or a community health worker (CHW)) [32]. In Togo, the situation is not different and health post at village level provide maternity services that are often run by matrones or other voluntary community health workers [60]. Institutional deliveries by unskilled attendants were also common – although not as much as in Togo and Senegal – in other African countries such as Benin (4 %), Central African Republic (5 %), and The Gambia (6 %).

In 1997, the World Health Organization document stated that “birth can take place in a range of appropriate settings, from home to tertiary referral centres, depending on availability and need [61]. It also recognized that home delivery could be appropriate for normal deliveries, provided the person attending the delivery is well trained and equipped [61]. Over time, the focus on SBA moved from coverage to quality of care in facilities, as many countries adopted strategies for promoting institutional deliveries. The World Health Report 2005 promoted care close to home – e.g. with midwives deployed in health centers and referral backup hospitals staffed by doctors, nurses and midwives [62]. Over time, the focus is also changing from measuring coverage to assessing and improving quality of care for facility births [63]. The shift from coverage to quality is at least in part motivated by studies showing that increased coverage of SBA and/or institutional deliveries does not necessarily improve maternal or newborn outcomes [64–68]. The growing focus on institutional birth should take into account the fact that in a few countries, many such deliveries are carried out by unskilled birth attendants, and therefore do not contribute to achieving the recommendations for increasing SBA coverage [18].

There is little evidence about the effectiveness of home SBA deliveries in the literature. Most research regarding home birth strategies has focused on the training of TBAs [15, 21], while home SBA deliveries have received little attention. In our study, pro-poor and pro-rural inequalities on home SBA deliveries at the national level were observed, consistently with previous publications [55, 69]. In spite of the existing inequalities among home SBA utilization, information on the quality of care provided at home remain scant. Constraints encountered by SBAs during home delivery are numerous and include inappropriate environment for delivery, insufficient supplies and equipment, lack of security, inadequate training for home delivery, lack of transportation for referrals, and the social pressure in life-and-death situations, all of which affect the quality of care [25, 70]. Some authors have stressed the limitations of home SBA deliveries for reducing maternal and newborn outcomes [51, 64]. On the other hand, several advantages have been reported for home SBAs deliveries, such as lower rate of medical interventions (episiotomy, forceps, vacuum extraction among others), social support, privacy and higher proportions of birth receiving skin-to-skin practices in immediate breastfeeding within one hour after birth [24, 71, 72]. Assessing the quality of home deliveries by SBAs is beyond the scope of the present analyses, but our contribution lies in highlighting that such births are common in several countries, and deserve further evaluation.

Our analyses have some limitations. The first is related to the definition of SBA. As initially proposed by WHO, SBAs included doctors, nurses and midwives [18]. In many countries, others cadres such as auxiliary nurses, auxiliary midwifes, community health workers, and even TBAs or matrones who received some degree of formal training may be considered as skilled, making it difficult to compare among countries [22]. We believe that analyses such as ours may help identify countries with unusual patterns, which may be associated with non-standard definitions of SBA. Second, SBA coverage in national surveys was based on maternal reports, and some women may be unable to provide accurate information. For example, Hussein et al. described the difficulties of women in discriminating accurately among different types of birth attendants in Ghana [73]. We believe that this situation could be similar in others countries, especially where SBA coverage is low. On the other hand, studies on the validity of self-report SBA question during delivery conducted in Kenya and Mexico have shown that, while this indicator is not recommended for use at the individual level, it could be used to generate acceptable estimate of SBA coverage at population level [74, 75]. Third, our analysis uses available data for countries for the period 2005–2014, but only three surveys were carried out prior to 2007, and with a few exceptions SBA coverage is increasing slowly in most countries [76]. Because not every country was included, there may be additional examples of the outlier patterns we described above. Fourth, wealth asset indices were used to analyze economic status; such indices may vary according to the choice of assets and are affected by issues of comparability between urban and rural household [77, 78]. In addition, wealth quintiles are relative measures, that is, the poorest quintile in a middle-income country might be the wealthier than the third or fourth quintile in an extremely poor country [27].

Nevertheless, use of asset indices allows systematic comparisons of inequalities in health that would not be possible with other more complex measures of socioeconomic position [27]. Finally, we defined inequality based on wealth asset indices and place of residence, a common tool for evaluating inequalities within populations [78]. Other determinants such as education, distance to health facility, ethnicity, occupation and religion, among others may be equally or even more important in affecting access to delivery care.

The above limitations, however, are unlikely to affect our main conclusions. To our knowledge, this is the first study that analyzes inequalities in these four categories of delivery assistance in a large number of LMICs countries using both DHS and MICS survey databases.

## Conclusions

We report a high correlation between coverage with SBA and institutional delivery coverage. As noted, approximately 98.0 % of institutional deliveries are performed by a SBA, and 96.0 % of SBA deliveries are in an institution. There are, however, some exceptions and 4,2 % of SBA deliveries are performed outside a health facility and 1,7 % of institutional deliveries are by unskilled birth attendants. Except for institutional deliveries carried out by SBAs, all other types of assistance were more common among the poor and rural populations. Analyses that take into account both place of delivery and type of attendant are important to help scale up safe delivery attendance for all women, and specially in remote areas where SBAs are scarce.

## Abbreviations

CAR, Central African Republic; CEE, Central and Eastern Europe; CHW, Community Health Worker; CIS, Commonwealth of Independent States; DHS, Demographic Health Surveys; FIGO, International Federation of Gynecology and obstetrics; ICM, International Confederation of Midwives; LAC, Latin American and Caribbean; LMIC, Low and Middle-Income Country; MDG, Millennium Development Goal; MICS, multiple indicator cluster surveys; SBA, skilled birth attendant; SD, standard deviation; TBA, traditional birth attendant; UNICEF, United Nations Children’s Fund; WHO, World Health Organization

## Additional files

Additional file 1: Web appendix A: DHS and MICS QUESTIONNAIRE. (PDF 178 kb) Additional file 2: (PDF 1141 kb) Additional file 3: Web appendix C: Distribution of place of delivery and type of professional in countries with at least 10 % of home SBA deliveries (Azerbaijan, Cambodia, Indonesia, Iraq, Philippines and Tajikistan) or at least 10 % of institutional deliveries by an unskilled worker (Senegal and Togo), by urban-rural residence. (PDF 217 kb) Additional file 4: Web appendix D: Distribution of place of delivery and type of professional in countries with at least 10 % of home SBA deliveries (Azerbaijan, Cambodia, Indonesia, Iraq, Philippines and Tajikistan) or at least 10 % of institutional deliveries by an unskilled worker (Senegal and Togo), by wealth quintile. (PDF 155 kb) Additional file 5: Web appendix B: ISO codes. (PDF 196 kb) Additional file 6: (XLSX 47 kb)
