# Supplementary material for: Inequalities in the coverage of place of delivery and skilled birth attendance: analyses of cross-sectional surveys in 80 low and middle-income countries
Source: Reprod Health. 2016 Jun 17;13:77. doi: 10.1186/s12978-016-0192-2 (PMC4912761; doi:10.1186/s12978-016-0192-2)
Supplement: Additional file 1: — Web appendix A: DHS and MICS QUESTIONNAIRE. (PDF 178 kb) [file 12978_2016_192_MOESM1_ESM.pdf]

Appendix A: DHS and MICS QUESTIONNAIRE

DHS QUESTIONNAIRE

429 (2)	Who assisted with the delivery of (NAME)?<br><br>Anyone else?<br><br>PROBE FOR THE TYPE(S) OF PERSON(S) AND RECORD ALL MENTIONED.<br><br>IF RESPONDENT SAYS NO ONE ASSISTED, PROBE TO DETERMINE WHETHER ANY ADULTS WERE PRESENT AT THE DELIVERY.	**HEALTH PERSONNEL**<br>DOCTOR . . . . A<br>NURSE/MIDWIFE . . . . B<br>AUXILIARY MIDWIFE . . . . C<br>**OTHER PERSON**<br>TRADITIONAL BIRTH ATTENDANT . . . . D<br>RELATIVE/FRIEND . . . . E<br>OTHER ____________ X<br>(SPECIFY)<br>NO ONE ASSISTED . . . . Y	**HEALTH PERSONNEL**<br>DOCTOR . . . . A<br>NURSE/MIDWIFE . . . . B<br>AUXILIARY MIDWIFE . . . . C<br>**OTHER PERSON**<br>TRADITIONAL BIRTH ATTENDANT . . . . D<br>RELATIVE/FRIEND . . . . E<br>OTHER ____________ X<br>(SPECIFY)<br>NO ONE ASSISTED . . . . Y

NO.	QUESTIONS AND FILTERS	LAST BIRTH<br>NAME ____________	NEXT-TO-LAST BIRTH<br>NAME ____________
430 (2)	Where did you give birth to (NAME)?<br><br>PROBE TO IDENTIFY THE TYPE OF SOURCE.<br><br>IF UNABLE TO DETERMINE IF PUBLIC OR PRIVATE SECTOR, WRITE THE NAME OF THE PLACE.<br><br>____________<br>(NAME OF PLACE)	**HOME**<br>HER HOME . . . . 11 (SKIP TO 434)<br>OTHER HOME . . . . 12 (SKIP TO 434)<br><br>**PUBLIC SECTOR**<br>GOVERNMENT HOSPITAL . . 21<br>GOVERNMENT HEALTH CENTER . . . . 22<br>GOVERNMENT HEALTH POST . . . . 23<br>OTHER PUBLIC SECTOR<br>____________ 26<br>(SPECIFY)<br><br>**PRIVATE MEDICAL SECTOR**<br>PRIVATE HOSPITAL/ CLINIC . . . . 31<br>OTHER PRIVATE MEDICAL SECTOR<br>____________ 36<br>(SPECIFY)<br><br>OTHER ____________ 96<br>(SPECIFY)<br>(SKIP TO 434)	**HOME**<br>HER HOME . . . . 11 (SKIP TO 434)<br>OTHER HOME . . . . 12 (SKIP TO 434)<br><br>**PUBLIC SECTOR**<br>GOVERNMENT HOSPITAL . . 21<br>GOVERNMENT HEALTH CENTER . . . . 22<br>GOVERNMENT HEALTH POST . . . . 23<br>OTHER PUBLIC SECTOR<br>____________ 26<br>(SPECIFY)<br><br>**PRIVATE MEDICAL SECTOR**<br>PRIVATE HOSPITAL/ CLINIC . . . . 31<br>OTHER PRIVATE MEDICAL SECTOR<br>____________ 36<br>(SPECIFY)<br><br>OTHER ____________ 96<br>(SPECIFY)<br>(SKIP TO 434)

MICS QUESTIONAIRE

**MN17**. WHO ASSISTED WITH THE DELIVERY OF (*name*)?<br><br>*Probe:*<br>ANYONE ELSE?<br><br>*Probe for the type of person assisting and circle all answers given.*<br><br>*If respondent says no one assisted, probe to determine whether any adults were present at the delivery.*	Health professional:<br>Doctor..A<br>Nurse / Midwife ....B<br>***Auxiliary midwife***.............................. C<br>Other person<br>Traditional birth attendant ....F<br>Community health worker .... G<br>Relative / Friend .... H<br><br>Other (*specify*)____________ X<br>No one ....Y	
**MN18**. WHERE DID YOU GIVE BIRTH TO (*name*)?<br><br>*Probe to identify the type of source.*<br><br>*If unable to determine whether public or private, write the name of the place.*<br><br>____________<br>*(Name of place)*	Home<br>Respondent's home .... 11<br>Other home .... 12<br><br>Public sector<br>Government hospital .... 21<br>Government clinic / health centre.......... 22<br>Government health post....................... 23<br>Other public (*specify*)____________ 26<br><br>Private Medical Sector<br>Private hospital.................................. 31<br>Private clinic .... 32<br>Private maternity home .... 33<br>Other private medical (*specify*)____________ 36<br><br>Other (*specify*)____________ 96	11⇨MN20<br>12⇨MN20<br><br><br><br><br><br><br><br><br><br><br><br><br><br><br>96⇨MN20
