# Supplementary material for: Inequalities in the coverage of place of delivery and skilled birth attendance: analyses of cross-sectional surveys in 80 low and middle-income countries
Source: Reprod Health. 2016 Jun 17;13:77. doi: 10.1186/s12978-016-0192-2 (PMC4912761; doi:10.1186/s12978-016-0192-2)

Web Table A1: Distribution of delivery assistance according to place of residence in LMICs (n=80 countries)

		SBA, institutional		Home, SBA		Institutional, non-SBA		Home, non-SBA		
**Country**	**Place of residence**	**N (%)**	**95% CI**	**N (%)**	**95% CI**	**N (%)**	**95% CI**	**N (%)**	**95% CI**	**p value**
Afghanistan 2010	urban	770(64.4)	61.7-67.1	113(9.5)	7.9-11.3	18(1.5)	1.0-2.4	294(24.6)	22.2-27.1	<0.001
	rural	882(25.3)	23.9-26.7	241(6.9)	6.1-7.8	38(1.1)	0.8-1.5	2329(66.7)	65.2-68.3	
Albania 2008	urban	384(98.7)	96.9-99.5	3(0.8)	0.2-2.4	0(0.0)	0.0-0.1	2(0.5)	0.1-2.0	0.019
	rural	469(95.7)	93.5-97.2	18(3.7)	2.3-5.8	0(0.0)	0.0-0.8	3(0.6)	0.2-1.9	
Armenia 2010	urban	600(99.3)	98.2-99.8	2(0.3)	0.1-1.3	0(0.0)	0.0-0.6	2(0.3)	0.1-1.3	0.329
	rural	334(100.0)	98.9-100.0	0(0.0)	0.0-1.1	0(0.0)	0.0-1.1	0(0.0)	0.0-1.1	
Azerbaijan 2006	urban	595(91.1)	88.7-93.1	30(4.6)	3.2-6.5	2(0.3)	0.1-1.2	26(4.0)	2.7-5.8	<0.001
	rural	544(72.6)	69.3-75.7	103(13.8)	11.5-16.4	2(0.3)	0.1-1.1	100(13.4)	11.1-16.0	
Bangladesh 2011	urban	755(48.4)	46.0-50.9	87(5.6)	4.5-6.8	10(0.6)	0.3-1.2	707(45.4)	42.9-47.8	<0.001
	rural	786(23.0)	21.6-24.5	103(3.0)	2.5-3.5	6(0.2)	0.1-0.4	2521(73.8)	72.3-75.2	
Belarus 2012	urban	987(99.8)	99.2-99.9	1(0.1)	0.0-0.7	0(0.0)	0.0-0.4	1(0.1)	0.0-0.7	0.712
	rural	335(100.0)	0.99-100.0	0(0.0)	0.0-1.1	0(0.0)	0.0-1.1	0(0.0)	0.0-1.1	
Belize 2011	urban	241(97.6)	94.7-98.9	2(0.8)	0.2-3.2	0(0.0)	0.0-1.5	4(1.6)	0.6-4.2	0.001
	rural	405(89.0)	85.8-91.6	22(4.8)	3.2-7.2	5(1.1)	0.5-2.6	23(5.1)	3.4-7.5	
Benin 2011	urban	2665(89.6)	88.4-90.6	19(0.6)	0.4-1.0	59(2.0)	1.5-2.6	233(7.8)	6.9-8.9	<0.001
	rural	3865(78.9)	77.8-80.1	61(1.3)	1.0-1.6	260(5.3)	4.7-6.0	710(14.5)	13.5-15.5	
Bhutan 2010	urban	448(87.6)	84.6-90.1	4(0.7)	0.3-1.9	6(1.1)	0.5-2.4	59(10.6)	8.3-13.4	<0.001
	rural	981(51.4)	49.2-53.7	60(3.1)	2.4-4.0	13(0.7)	0.4-1.2	854(44.8)	42.5-47.0	
Bolivia 2008	urban	2397(89.3)	88.0-90.4	22(0.8)	0.5-1.2	13(0.5)	0.3-0.8	253(9.4)	8.4-10.6	<0.001
	rural	1289(50.8)	48.8-52.7	168(6.6)	5.7-7.6	6(0.2)	0.1-0.5	1077(42.4)	40.5-44.3	

Bosnia & Herz. 2011	urban	242(99.6)	97.1-99.9	1(0.4)	0.1-2.9	0(0.0)	0.0-1.5	0(0.0)	0.0-1.5	0.689
	rural	437(99.6)	98.3-99.9	1(0.2)	0.0-1.5	0(0.0)	0.0-0.8	1(0.2)	0.0-1.5	
Brazil 2006	urban	1872(96.0)	95.0-96.8	5(0.3)	0.1-0.6	30(1.5)	1.1-2.2	43(2.2)	1.6-3.0	<0.001
	rural	929(93.2)	91.4-94.6	4(0.4)	0.2-1.1	12(1.2)	0.7-2.1	52(5.2)	4.0-6.8	
Burkina Faso 2010	urban	1856(93.4)	92.2-94.4	7(0.4)	0.2-0.7	5(0.5)	0.1-0.6	119(6.0)	5.0-7.1	<0.001
	rural	4735(67.5)	66.4-68.6	70(1.0)	0.8-1.3	12(0.2)	0.1-0.3	2198(31.3)	30.3-32.4	
Burundi 2010	urban	745(89.2)	86.9-91.2	13(1.6)	0.9-2.7	0(0.0)	0.0-0.4	77(9.2)	7.4-11.4	<0.001
	rural	2431(62.1)	60.6-63.6	33(0.8)	0.6-1.2	15(0.4)	0.2-0.6	1436(36.7)	35.2-38.2	
Cambodia 2010	urban	1057(83.1)	80.9-85.1	129(10.1)	8.6-11.9	5(0.4)	0.2-0.9	81(6.4)	5.2-7.9	<0.001
	rural	1907(51.8)	49.8-53.0	537(14.5)	13.4-15.7	10(0.3)	0.1-0.5	1254(33.8)	32.3-35.4	
Cameroon 2011	urban	2463(85.1)	83.7-86.3	76(2.6)	2.1-3.3	10(0.4)	0.2-0.6	347(12.0)	10.8-13.2	<0.001
	rural	2116(48.8)	47.3-50.3	136(3.1)	2.7-3.7	13(0.3)	0.2-0.5	2071(47.8)	46.3-49.3	
CAR 2010	urban	1025(70.9)	68.5-73.2	99(6.9)	5.7-8.3	46(3.2)	2.4-4.2	276(19.1)	17.1-21.2	<0.001
	rural	1108(35.8)	34.1-37.5	190(6.1)	5.3-7.0	258(8.3)	7.4-9.4	1543(49.8)	48.0-51.6	
Chad 2010	urban	829(33.8)	32.0-35.7	308(12.6)	11.3-13.9	18(0.7)	0.5-1.2	1297(52.9)	50.9-54.9	<0.001
	rural	250(6.8)	6.0-7.6	190(5.1)	4.5-5.9	13(0.4)	0.2-1.6	3248(87.8)	86.7-88.8	
Comoros 2012	urban	600(89.3)	86.7-91.4	27(4.0)	2.8-5.8	1(0.2)	0.0-1.1	44(6.6)	4.9-8.7	<0.001
	rural	900(70.4)	67.9-72.9	96(7.5)	6.2-9.1	4(0.3)	0.1-0.8	278(21.8)	19.6-24.1	
Congo Brazzaville 2011	urban	1483(95.7)	94.6-96.6	29(1.9)	1.3-2.7	4(0.3)	0.1-0.7	33(2.1)	1.5-3.0	<0.001
	rural	3449(81.4)	80.2-82.6	186(4.4)	3.8-5.1	14(0.3)	0.2-0.6	587(13.9)	19.6-24.1	
Congo D.R. 2013	urban	3067(90.2)	89.1-91.1	59(1.7)	1.3-2.2	35(1.0)	0.7-1.4	240(7.1)	6.2-8.0	<0.001
	rural	5192(64.9)	63.8-65.9	230(2.9)	2.5-3.3	221(2.8)	2.4-3.1	2358(29.5)	28.5-30.5	
Costa Rica 2011	urban	426(99.1)	97.5-99.7	0(0.0)	0.0-0.9	3(0.7)	0.2-2.1	1(0.2)	0.0-1.6	0.007
	rural	408(96.2)	93.9-97.7	6(1.4)	0.6-3.1	2(0.5)	0.1-1.9	8(1.9)	0.9-3.7	
	urban	1297(80.2)	78.2-82.1	33(2.0)	1.5-2.9	6(0.4)	0.2-0.8	281(17.38)	15.6-19.3	<0.001

Cote d'Ivoire 2011	rural	1361(42.7)	41.0-44.4	77(2.4)	1.9-3.0	32(1.0)	0.7-1.4	1719(53.9)	52.2-55.6	
Dominican Republic 2013	urban	1525(98.2)	97.4-98.8	5(0.3)	0.1-0.8	9(0.6)	0.3-1.1	14(0.9)	0.5-1.5	<0.001
	rural	630(95.2)	93.2-96.6	5(0.8)	0.3-1.8	3(0.5)	0.1-1.4	24(3.6)	2.4-5.4	
Egypt 2008	urban	2195(85.5)	84.1-86.9	132(5.1)	4.4-6.1	6(0.2)	0.1-0.5	233(9.1)	8.0-10.3	<0.001
	rural	2780(64.5)	63.1-65.9	369(8.6)	7.8-9.4	10(0.2)	0.1-0.4	1151(26.7)	25.4-28.0	
Ethiopia 2011	urban	728(61.2)	58.4-63.9	19(1.6)	1.0-2.5	2(0.2)	0.0-0.7	441(37.1)	34.4-39.8	<0.001
	rural	258(4.7)	4.2-5.3	40(0.8)	0.5-1.0	9(0.2)	0.1-0.3	5204(94.4)	93.8-95.6	
Gabon 2012	urban	2193(91.8)	90.6-92.8	17(0.7)	0.4-1.1	16(0.7)	0.4-1.1	163(6.8)	5.9-7.9	<0.001
	rural	975(66.2)	63.7-68.5	20(1.4)	0.9-2.1	16(1.1)	0.7-1.8	463(31.4)	29.1-33.8	
Gambia 2013	urban	1353(78.0)	76.0-79.9	12(0.7)	0.4-1.2	129(7.4)	6.3-8.8	240(13.8)	12.3-15.5	<0.001
	rural	1335(39.2)	37.6-40.9	39(1.2)	0.8-1.6	185(5.4)	4.7-6.2	1845(54.2)	52.5-55.9	
Ghana 2011	urban	661(83.6)	80.8-86.0	7(0.9)	0.4-1.8	2(0.3)	0.1-1.0	121(15.3)	13.0-18.0	<0.001
	rural	922(44.3)	42.2-46.4	29(1.4)	1.0-2.0	15(1.4)	0.4-1.2	1116(53.6)	51.5-55.7	
Guatemala 2008	urban	1786(75.1)	73.3-76.8	14(0.6)	0.3-1.0	10(0.4)	0.2-0.8	568(23.9)	22.2-25.6	<0.001
	rural	1598(38.7)	37.3-40.2	19(0.5)	0.3-0.7	17(0.4)	0.3-0.7	2492(60.4)	58.9-61.9	
Guinea 2012	urban	854(67.8)	65.2-70.4	184(14.6)	12.8-16.7	5(0.4)	0.2-1.0	216(17.2)	15.2-19.3	<0.001
	rural	770(25.9)	24.3-27.5	117(3.9)	3.3-4.7	62(2.1)	1.6-2.7	2027(68.1)	66.4-69.8	
Guyana 2009	urban	241(98.0)	95.2-99.2	4(1.6)	0.6-4.3	1(0.4)	0.1-2.8	0(0.0)	0.0-1.5	<0.001
	rural	879(75.7)	73.2-78.1	81(7.0)	5.6-8.6	11(1.0)	0.5-1.7	190(16.4)	14.3-18.6	
Haiti 2012	urban	881(57.2)	54.8-59.7	37(2.4)	1.7-3.3	5(0.3)	0.1-0.8	616(40.0)	37.6-42.5	<0.001
	rural	701(23.4)	21.9-24.9	40(1.3)	1.0-1.8	12(0.4)	0.2-0.7	2248(74.9)	73.3-76.4	
Honduras 2011	urban	2158(94.0)	93.0-94.9	15(0.7)	0.4-1.1	3(0.1)	0.0-0.4	119(5.2)	4.3-6.2	<0.001
	rural	3216(72.6)	91.3-93.9	13(0.3)	0.2-0.5	15(0.3)	0.2-0.6	1185(26.8)	25.5-28.1	
India 2005	urban	7928(69.1)	68.3-70.0	728(6.4)	5.9-6.8	41(0.4)	0.3-0.5	2773(24.2)	23.4-25.0	<0.001
	rural	6249(33.0)	32.3-33.7	1717(9.1)	8.7-9.5	48(0.3)	0.2-0.3	10918(57.7)	57.0-58.4	

Indonesia 2012	urban	3914(79.0)	77.9-80.1	686(13.9)	12.9-14.8	13(0.3)	0.2-0.5	340(6.9)	6.2-7.6	<0.001
	rural	2362(39.6)	38.4-40.9	1832(30.7)	29.6-31.9	5(0.1)	0.0-0.2	1760(29.5)	28.4-30.7	
Iraq 2011	urban	6097(78.4)	77.5-79.3	1079(13.9)	13.1-14.7	53(0.7)	0.5-0.9	549(7.1)	6.4-7.6	<0.001
	rural	4164(67.0)	65.8-68.1	859(13.8)	13.0-14.7	18(0.3)	0.2-0.5	1175(18.9)	17.9-19.9	
Jamaica 2011	urban	378(99.0)	97.2-99.6	3(0.8)	0.3-2.4	1(0.3)	0.0-1.8	0(0.0)	0.0-1.0	0.077
	rural	242(97.6)	94.7-98.9	2(0.8)	0.2-3.2	0(0.0)	0.0-1.5	4(1.6)	0.6-4.2	
Jordan 2012	urban	4136(99.1)	98.7-99.3	27(0.7)	0.4-0.9	1(0.0)	0.0-0.2	11(0.3)	0.1-0.5	0.124
	rural	1934(99.6)	99.2-99.8	4(0.2)	0.1-0.5	0(0.0)	0.0-0.2	4(0.2)	0.1-0.5	
Kazakhstan 2010	urban	1066(99.7)	99.1-99.9	0(0.0)	0.0-0.3	0(0.0)	0.0-0.3	3(0.3)	0.1-0.9	0.028
	rural	954(99.6)	98.9-99.8	4(0.4)	0.2-1.1	0(0.0)	0.0-0.4	0(0.0)	0.0-0.4	
Kenya 2008	urban	679(74.3)	71.4-77.0	10(1.1)	0.6-2.0	5(0.6)	0.2-1.3	220(24.1)	21.4-27.0	<0.001
	rural	951(33.7)	32.0-35.5	77(2.7)	2.2-3.4	1(0.0)	0.0-0.3	1790(63.5)	61.7-65.3	
Kyrgyzstan 2012	urban	705(99.6)	98.7-99.9	0(0.0)	0,0-0.5	0(0.0)	0.0-0.5	3(0.4)	0.1-1.3	0.238
	rural	2029(98.8)	98.3-99.2	9(0.4)	0.2-0.8	2(0.1)	0.0-0.4	13(0.6)	0.4-1.1	
Lao 2011	urban	631(70.9)	67.8-73.8	50(5.6)	4.3-7.3	1(0.1)	0.0-0.8	208(23.4)	20.7-26.3	<0.001
	rural	865(24.3)	23.0-25.8	132(3.7)	3.1-4.4	23(0.7)	0.4-1.0	2534(71.3)	69.8-72.8	
Lesotho 2009	urban	356(86.0)	82.3-89.0	14(3.4)	2.0-5.6	3(0.7)	0.2-2.2	41(9.9)	7.4-13.2	<0.001
	rural	1044(49.8)	47.7-52.0	63(3.0)	2.4-3.8	6(0.3)	0.1-0.6	983(46.9)	44.8-49.0	
Liberia 2013	urban	998(68.4)	66.0-70.7	83(5.7)	4.6-7.0	20(1.4)	0.9-2.1	358(24.5)	22.4-26.8	<0.001
	rural	1485(47.4)	45.5-49.1	153(4.9)	4.2-5.7	70(2.2)	1.8-2.8	1428(45.5)	43.8-47.3	
Macedonia 2011	urban	259(98.1)	95.5-99.2	2(0.8)	0.2-3.0	3(1.1)	0.4-3.5	0(0.0)	0.0-1.4	0.067
	rural	233(97.5)	94.5-98.9	2(0.8)	0.2-3.3	0(0.0)	0.0-1.5	4(1.7)	0.6-4.4	
Madagascar 2008	urban	786(60.5)	57.8-63.1	260(20.0)	17.9-22.3	3(0.2)	0.1-0.7	251(19.3)	17.3-21.5	<0.001
	rural	1853(30.3)	29.2-31.5	419(6.9)	6.2-7.5	13(0.2)	0.1-0.4	3830(62.6)	61.4-63.8	
Malawi 2010	urban	993(88.2)	86.2-89.9	4(0.4)	0.1-0.9	23(2.0)	1.4-3.1	106(9.4)	7.8-11.3	<0.001
	rural	7791(70.8)	69.9-71.6	44(0.4)	0.3-0.5	250(2.3)	2.0-2.6	2925(26.6)	25.7-27.4	
	urban	312(97.8)	95.5-99.0	6(1.9)	0.8-4.1	0(0.0)	0.0-1.2	1(0.3)	0.0-2.2	0.001

Maldives 2009	rural	2013(94.8)	93.7-95.6	17(0.8)	0.5-1.3	32(1.5)	1.1-2.1	62(2.9)	2.3-3.7	
Mali 2012	urban	1412(92.5)	91.1-93.7	19(1.3)	0.8-1.9	9(0.6)	0.3-1.1	86(5.6)	4.6-6.9	<0.001
	rural	2059(45.9)	44.4-47.3	222(4.9)	4.3-5.6	51(1.1)	0.9-1.5	2158(48.1)	46.6-49.5	
Moldova 2012	urban	403(99.50	98.0-99.9	0(0.0)	0.0-0.9	0(0.0)	0.0-0.9	2(0.5)	0.1-2.0	0.213
	rural	313(98.4)	96.3-99.3	2(0.6)	0.2-2.5	0(0.0)	0.0-1.2	3(0.9)	0.3-2.9	
Mongolia 2010	urban	773(99.2)	98.3-99.7	3(0.4)	0.1-1.2	0(0.0)	0.0-0.5	3(0.3)	0.1-1.2	0.03
	rural	889(97.6)	96.4-98.4	4(0.4)	0.2-1.2	3(0.3)	0.1-1.0	15(1.7)	1.0-2.7	
Montenegro 2013	urban	315(99.4)	97.5-99.8	0(0.0)	0.0-1.2	0(0.0)	0.0-1.2	2(0.6)	0.2-2.5	0.928
	rural	176(99.4)	96.1-99.9	0(0.0)	0.0-2.1	0(0.0)	0.0-2.1	1(0.5)	0.1-3.9	
Mozambique 2011	urban	1867(82.8)	81.2-84.3	14(0.6)	0.4-1.0	65(2.9)	2.3-3.7	308(13.7)	12.3-15.1	<0.001
	rural	2334(49.8)	48.4-51.3	82(1.8)	1.4-2.2	117(2.5)	2.1-3.0	2150(45.9)	44.5-47.3	
Namibia 2013	urban	1325(94.1)	92.7-95.2	10(0.7)	0.4-1.3	6(0.4)	0.2-0.9	67(4.8)	3.8-6.0	<0.001
	rural	1380(78.7)	76.7-80.6	38(2.2)	1.6-3.0	5(0.3)	0.1-0.7	330(18.8)	17.1-20.7	
Nepal 2011	urban	460(72.4)	68.8-75.8	13(2.1)	1.2-3.5	3(0.5)	0.2-1.5	159(25.0)	21.8-28.6	<0.001
	rural	790(32.0)	30.2-33.9	27(1.1)	0.8-1.6	45(1.8)	1.4-2.4	1605(65.1)	63.2-66.9	
Niger 2012	urban	1471(86.0)	84.3-87.6	15(0.9)	0.5-1.5	7(0.4)	0.2-0.9	217(12.7)	11.2-14.4	<0.001
	rural	1362(23.5)	22.4-24.6	41(0.7)	0.5-1.0	83(1.4)	1.2-1.8	4319(74.4)	73.3-75.5	
Nigeria 2013	urban	4006(63.9)	62.7-65.1	369(5.9)	5.3-6.5	80(1.3)	1.0-1.6	1813(28.9)	27.8-30.1	<0.001
	rural	2807(22.3)	21.6-23.0	329(2.6)	2.3-2.9	286(2.3)	2.0-2.5	9179(72.8)	72.1-73.1	
Pakistan 2012	urban	1917(66.8)	65.0-68.5	98(3.4)	2.8-4.1	15(0.5)	0.3-0.9	841(29.3)	27.7-31.0	<0.001
	rural	1720(43.2)	41.7-44.8	183(4.6)	4.0-5.3	21(0.5)	0.3-0.8	2054(51.6)	50.1-53.2	
Peru 2012	urban	3184(94.8)	94.0-95.5	30(0.9)	0.6-1.3	9(0.3)	0.1-0.5	136(4.1)	3.4-4.8	<0.001
	rural	1573(67.0)	65.0-68.8	112(4.8)	4.0-5.7	28(1.2)	0.8-1.7	636(27.1)	25.3-28.9	
Philippines 2013	urban	1296(74.4)	72.3-76.4	146(8.4)	7.2-9.8	1(0.1)	0.0-0.4	298(17.1)	15.4-19.0	<0.001
	rural	1373(54.0)	53.6-56.5	238(10.0)	4.4-10.7	6(0.2)	0.1-0.5	899(35.7)	33.9-37.6	
	urban	595(85.7)	82.9-88.1	5(0.7)	0.3-1.7	3(0.4)	0.1-1.3	91(13.1)	10.8-15.8	<0.001

Rwanda 2010	rural	3391(75.3)	74.0-76.5	14(0.3)	0.2-0.5	14(0.3)	0.2-0.5	1086(24.1)	22.9-25.4	
São Tome & Principe 2008	urban	397(84.8)	81.3-87.8	17(3.6)	2.3-5.8	0(0.0)	0.0-0.8	54(11.5)	8.9-14.8	<0.001
	rural	542(73.9)	70.6-77.0	27(3.7)	2.5-5.3	4(0.6)	0.2-1.4	160(21.8)	19.0-25.0	
Senegal 2014	urban	932(73.9)	71.4-76.2	22(1.7)	1.2-2.6	201(15.9)	14.0-18.1	107(8.5)	7.1-10.1	<0.001
	rural	1159(40.3)	38.6-42.1	16(0.6)	0.3-0.9	611(21.3)	19.8-22.8	1087(37.8)	36.1-39.6	
Serbia 2014	urban	595(99.2)	98.0-99.7	1(0.2)	0.0-1.2	0(0.0)	0.0-0.6	4(0.7)	0.2-1.8	0.711
	rural	354(98.6)	96.7-99.4	1(0.3)	0.0-2.0	0(0.0)	0.0-1.0	4(1.11)	0.4-2.9	
Sierra Leone 2013	urban	1558(72.9)	71.0-74.8	198(9.3)	8.1-10.6	20(0.9)	0.6-1.4	360(16.9)	15.3-18.5	<0.001
	rural	2572(51.9)	50.5-53.3	222(4.5)	3.9-5.1	38(0.8)	0.6-1.1	2127(42.9)	41.5-44.3	
St Lucia 2012	urban	33(100.0)	89.4-100.0	0(0.0)	0.0-10.6	0(0.0)	0.0-10.6	0(0.0)	0.0-10.6	0.474
	rural	64(98.5)	89.5-99.8	0(0.0)	0.0-5.5	1(1.5)	0.2-10.5	0(0.0)	0.0-5.5	
State of Palestine 2010	urban	3498(98.4)	97.9-98.8	40(1.1)	0.8-1.5	16(0.5	0.0-0.3	25(0.7)	0.2-0.6	0.072
	rural	755(97.4)	96.0-98.3	12(1.6)	0.9-2.7	10(1.3)	0.2-1.4	10(1.3)	0.2-1.4	
Suriname 2010	urban	375(93.1)	90.1-95.2	14(3.5)	2.1-5.8	6(1.5)	0.7-3.3	8(2.0)	1.0-3.9	<0.001
	rural	678(78.7)	75.8-81.3	54(6.3)	4.8-8.1	81(9.4)	7.6-11.5	49(5.7)	4.3-7.4	
Swaziland 2010	urban	277(85.5)	81.2-88.9	10(3.1)	1.7-5.6	3(1.0)	0.3-2.8	34(10.5)	7.6-14.3	0.001
	rural	535(77.1)	73.8-80.1	11(1.6)	0.9-2.8	9(1.3)	0.7-2.5	139(20.0)	17.2-23.2	
Tajikistan 2012	urban	857(88.7)	86.6-90.6	58(6.0)	4.7-7.7	3(0.3)	0.1-1.0	48(5.0)	3.8-6.5	<0.001
	rural	1649(74.0)	72.1-75.8	294(13.2)	11.8-14.7	10(0.5)	0.2-0.8	276(12.4)	11.1-13.8	
Tanzania 2010	urban	722(79.1)	76.3-81.6	22(2.4)	1.6-3.6	6(0.7)	0.3-1.5	163(17.9)	15.5-20.5	<0.001
	rural	1604(40.9)	39.4-42.5	59(1.5)	1.2-1.9	28(0.7)	0.5-1.0	2229(56.9)	55.3-58.4	
	urban	580(44.4)	41.8-47.2	89(6.8)	5.6-8.3	4(0.3)	0.1-0.8	632(48.4)	45.7-51.1	<0.001

Timor Leste 2009	rural	665(14.5)	13.5-15.5	410(8.9)	8.1-9.8	5(0.1)	0.0-0.3	3521(76.5)	75.3-77.7	
Togo 2013	urban	1130(91.9)	90.3-93.3	9(0.7)	0.4-1.4	35(2.9)	2.1-3.9	55(4.5)	3.5-5.8	<0.001
	rural	1199(39.8)	38.0-41.5	3(0.1)	0.0-0.3	639(21.2)	19.8-22.7	1174(38.9)	37.2-40.7	
Tunisia 2011	urban	642(99.4)	98.4-99.8	0(0.0)		0(0.0)	0.0-0.6	4(0.6)	0.2-1.6	<0.001
	rural	462(94.5)	92.1-96.2	2(0.4)	0.1-1.6	0(0.0)	0.0-0.8	25(5.1)	3.5-7.5	
Uganda 2011	urban	909(88.8)	86.7-90.6	15(1.5)	0.9-2.4	9(0.9)	0.5-1.7	91(8.9)	7.3-10.8	<0.001
	rural	1912(51.2)	49.6-52.8	69(1.9)	1.5-2.3	54(1.5)	1.1-1.9	1702(45.5)	44.0-47.1	
Ukraine 2012	urban	981(99.1)	98.3-99.5	2(0.2)	0.1-0.8	0(0.0)	0.0-0.4	7(0.7)	0.3-1.5	0.50
	rural	565(98.4)	97.0-99.2	2(0.4)	0.1-1.4	0(0.0)	0.0-0.6	7(1.2)	0.6-2.5	
Vietnam 2010	urban	530(97.8)	96.1-98.7	3(0.6)	0.2-1.7	0(0.0)	0.0-0.7	9(1.7)	0.9-3.2	<0.001
	rural	684(83.3)	80.6-85.7	11(1.3)	0.7-2.4	5(0.6)	0.3-1.5	121(14.7)	12.5-17.3	
Zambia 2007	urban	1039(79.9)	77.6-82.0	5(0.4)	0.2-0.9	15(1.2)	0.7-1.9	242(18.6)	16.6-20.8	<0.001
	rural	851(31.7)	30.0-33.5	10(0.4)	0.2-0.7	56(2.1)	1.6-2.7	1766(65.8)	64.0-67.6	
Zimbabwe 2010	urban	902(85.8)	83.6-87.8	12(1.1)	0.6-2.0	2(0.2)	0.0-0.8	135(12.8)	11.0-15.0	<0.001
	rural	1407(55.5)	53.2-57.1	54(2.1)	1.6-2.8	21(0.8)	0.5-1.3	1068(41.9)	40.0-43.8	

p: Chi-square test of heterogeneity; SBA: skilled birth attendant. CAR: Central African Republic

Web Table A2: Distribution of delivery assistance according to economic status in LMICs (n=80 countries)

		SBA, institutional		Home, SBA		Institutional, non-SBA		Home, non-SBA		
**Country**	**Wealth quintile**	**N (%)**	**95% CI**	**N (%)**	**95% CI**	**N (%)**	**95% CI**	**N (%)**	**95% CI**	**p value**
Afghanistan 2010	Q1	103(12.6)	10.3-14.9	35(4.3)	3.0-5.7	6(0.7)	0.1-1.3	673(82.4)	79.8-85.0	<0.001
	Q2	173(18.5)	16.0-21.0	58(6.2)	4.7-7.8	13(1.4)	0.6-2.1	690(93.9)	71.1-76.7	
	Q3	261(28.9)	25.9-31.8	50(5.5)	4.4-7.0	14(1.6)	0.7-2.4	579(64.1)	61.0-67.2	
	Q4	366(39.6)	36.5-42.8	97(10.5)	8.5-12.5	9(0.9)	0.3-1.6	452(48.9)	45.7-52.1	
	Q5	749(67.7)	65.0-70.5	114(10.3)	8.5-12.1	14(1.3)	0.6-1.9	229(20.7)	18.3-23.1	
Albania 2008	Q1	196(91.6)	87.9-95.3	16(7.5)	3.9-11.0	0(0.0)	0.0-1.7	2(0.9)	0.4-2.2	<0.001
	Q2	158(98.1)	96.0-100.0	2(1.2)	0.5-3.0	0(0.0)	0.0-2.3	1(0.6)	0.6-1.8	
	Q3	178(99.0)	97.4-100.0	0(0.0)	0.0-2.0	0(0.0)	0.0-2.0	2(1.1)	0.4-2.6	
	Q4	176(99.0)	97.3-100.0	2(1.1)	0.4-3.0	0(0.0)	0.0-2.0	0(0.0)	0.0-2.0	
	Q5	145(99.3)	98.0-100.0	1(0.7)	0.7-2.0	0(0.0)	0.0-2.5	0(0.0)	0.0-2.5	
Armenia 2010	Q1	165(100.0)	97.8-100.0	0(0.0)	0.0-2.2	0(0.0)	0.0-2.2	0(0.0)	0.0-2.2	0.667
	Q2	205(99.5)	98.6-100.0	0(0.0)	0.0-2.8	0(0.0)	0.0-2.8	1(0.5)	0.5-1.4	
	Q3	203(100.0)	98.2-100.0	0(0.0)	0.0-1.8	0(0.0)	0.0-1.8	0(0.0)	0.0-1.8	
	Q4	221(99.10)	97.7-100.0	1(0.5)	0.4-1.3	0(0.0)	0.0-1.6	1(0.5)	0.4-1.3	
	Q5	140(99.3)	98.0-100.0	1(0.7)	0.7-2.1	0(0.0)	0.0-2.6	0(0.0)	0.0-2.6	

Azerbaijan 2006	Q1	221(66.6)	61.5-71.7	46(13.86)	10.1-17.6	3(0.9)	0.1-1.9	62(18.7)	14.5-22.9	<0.001
	Q2	260(74.90	70.4-79.5	51(14.7)	11.0-18.4	1(0.3)	0.3-0.9	35(10.1)	6.9-13.3	
	Q3	275(84.6)	80.7-88.5	27(8.3)	5.3-11.3	0(0.0)	0.0-1.1	23(7.1)	4.3-9.9	
	Q4	220(94.0)	91.0-97.1	8(3.4)	1.1-5.8	0(0.0)	0.0-1.6	6(2.6)	0.5-4.6	
	Q5	163(99.4)	98.2-100.0	1(0.6)	0.6-1.8	0(0.0)	0.0-2.2	0(0.0)	0.0-2.2	
Bangladesh 2011	Q1	124(11.6)	9.7-13.5	22(2.1)	1.2-2.9	1(0.1)	0.1-0.3	923(86.3)	84.2-88.3	<0.001
	Q2	164(17.2)	14.8-19.6	18(1.9)	1.0-2.7	4(0.4)	0.0-0.8	769(80.5)	78.0-83.0	
	Q3	243(25.7)	22.9-28.5	46(4.9)	3.5-6.2	1(0.1)	0.1-0.3	655(69.3)	66.4-72.3	
	Q4	391(39.0)	36.0-42.0	51(5.1)	3.7-6.5	5(0.5)	0.1-0.9	555(53.4)	52.3-58.5	
	Q5	619(61.7)	58.7-64.7	53(5.3)	3.9-6.7	5(0.5)	0.1-0.9	326(32.5)	29.6-35.4	
Belarus 2012	Q1	177(100.0)	97.9-100.0	0(0.0)	0.0-2.1	0(0.0)	0.0-2.1	0(0.0)	0.0-2.1	0.412
	Q2	245(100.0)	98.5-100.0	0(0.0)	0.0-1.5	0(0.0)	0.0-1.5	0(0.0)	0.0-1.5	
	Q3	235(99.6)	98.7-100.0	0(0.0)	0.0-1.6	0(0.0)	0.0-1.6	1(0.4)	0.4-1.3	
	Q4	286(99.6)	98.9-100.0	1(0.4)	0.3-1.0	0(0.0)	0.0-1.3	0(0.0)	0.0-1.3	
	Q5	379(100.0)	99.0-100.0	0(0.0)	0.0-1.0	0(0.0)	0.0-1.0	0(0.0)	0.0-1.0	
Belize 2011	Q1	171(80.7)	75.3-86.0	17(8.0)	4.3-11.7	4(1.9)	0.0-3.7	20(9.4)	5.5-13.4	<0.001
	Q2	148(98.0)	95.8-100.0	0(0.0)	0.0-2.4	1(0.7)	0.6-2.0	2(1.3)	0.5-3.2	
	Q3	127(95.5)	91.9-99.0	4(3.0)	0.1-5.9	0(0.0)	0.0-2.7	2(1.5)	0.6-3.6	
	Q4	123(97.6)	94.9-100.0	2(1.59)	0.6-3.8	0(0.0)	0.0-2.9	1(0.8)	0.7-2.4	
	Q5	77(96.3)	92.1-100.0	1(1.3)	1.2-3.7	0(0.0)	0.0-4.5	2(2.5)	0.9-5.9	
Benin 2011	Q1	1137(63.7)	61.5-65.9	27(1.5)	0.9-2.1	138(7.7)	6.5-9.0	483(27.1)	25.0-29.1	<0.001
	Q2	1325(78.6)	76.6-80.5	31(1.8)	1.2-2.5	87(5.2)	4.1-6.2	243(14.4)	12.7-16.1	
	Q3	1396(84.7)	83.0-86.4	14(0.9)	0.4-1.3	67(4.1)	3.1-5.0	171(10.4)	8.9-11.8	
	Q4	1458(96.1)	95.1-97.0	4(0.3)	0.0-0.5	21(1.4)	0.8-2.0	35(2.3)	1.6-3.1	
	Q5	1214(98.3)	97.5-99.0	4(0.3)	0.0-0.6	6(0.5)	0.0-0.9	11(0.9)	0.4-1.4	
Bhutan 2010	Q1	160(31.3)	27.3-35.3	14(2.7)	1.3-4.2	5(1.0)	0.1-1.8	332(65.0)	60.8-69.1	<0.001
	Q2	223(41.8)	37.6-46.0	17(3.2)	1.7-4.7	3(0.6)	0.0-1.2	290(54.4)	50.2-58.6	

	Q3	326(61.8)	57.7-66.0	19(3.6)	2.0-5.2	2(0.4)	0.1-0.9	180(34.2)	30.1-38.2	
	Q4	384(77.6)	73.9-81.3	13(2.6)	1.2-4.0	6(1.2)	0.2-2.2	92(18.6)	15.2-22.0	
	Q5	376(94.2)	91.9-96.5	1(0.3)	0.2-0.7	3(0.8)	0.1-1.6	19(4.8)	2.7-6.9	
Bolivia 2008	Q1	558(37.7)	35.2-40.1	106(7.2)	5.8-8.5	3(0.2)	0.0-0.4	814(55.0)	52.4-57.5	<0.001
	Q2	766(67.4)	64.6-70.1	64(5.6)	4.3-7.0	1(0.1)	0.1-0.3	306(26.9)	24.3-29.5	
	Q3	886(83.6)	81.4-85.8	16(1.5)	0.8-2.2	6(0.6)	0.1-1.0	152(14.3)	12.2-16.5	
	Q4	832(93.1)	91.4-94.7	3(0.3)	0.0-0.7	7(0.8)	0.2-1.4	52(5.8)	4.3-7.4	
	Q5	644(98.6)	97.7-99.5	1(0.2)	0.1-0.5	2(0.3)	0.1-0.7	6(0.9)	0.2-1.7	
Bosnia & Herz. 2011	Q1	112(100.0)	96.8-100.0	0(0.0)	0.0-3.2	0(0.0)	0.0-3.2	0(0.0)	0.0-3.2	0.584
	Q2	148(98.7)	96.8-100.0	1(0.7)	0.6-2.0	1(0.7)	0.0-2.4	1(0.7)	0.6-2.0	
	Q3	151(99.3)	98.1-100.0	1(0.7)	0.6-1.9	0(0.0)	0.0-2.4	0(0.0)	0.0-2.4	
	Q4	139(100.0)	97.4-100.0	0(0.0)	0.0-2.6	0(0.0)	0.0-2.6	0(0.0)	0.0-2.6	
	Q5	165(100.0)	97.8-100.0	0(0.0)	0.0-2.2	0(0.0)	0.0-2.8	0(0.0)	0.0-2.8	
Brazil 2006	Q1	1046(91.27)	89.6-92.9	3(0.3)	0.0-0.6	20(1.8)	1.0-2.5	77(6.8)	5.3-8.2	<0.001
	Q2	731(96.6)	95.3-97.9	1(0.1)	0.1-0.4	12(1.6)	0.7-2.5	13(1.7)	0.8-2.6	
	Q3	468(97.5)	96.1-98.9	2(0.4)	0.2-1.0	6(1.3)	0.3-2.2	4(0.8)	0.0-1.6	
	Q4	332(98.2)	96.8-99.6	1(0.3)	0.3-0.8	4(1.2)	0.0-2.3	1(0.3)	0.3-0.9	
	Q5	224(99.1)	97.9-100.0	2(0.9)	0.3-2.1	0(0.0)	0.0-1.6	0(0.0)	0.0-1.6	
Burkina Faso 2010	Q1	886(52.2)	49.8-54.6	10(0.6)	0.2-1.0	4(0.2)	0.0-0.5	797(47.0)	44.6-49.3	<0.001
	Q2	1200(63.9)	61.7-66.1	28(1.5)	1.0-2.0	5(0.3)	0.0-0.5	645(34.4)	32.2-36.5	
	Q3	1484(74.0)	72.1-75.9	21(1.1)	0.6-1.5	2(0.1)	0.0-0.2	498(24.8)	22.9-26.7	
	Q4	1634(92.2)	81.5-84.8	15(0.8)	0.4-1.1	2(0.1)	0.0-0.2	314(16.0)	14.4-17.6	
	Q5	1387(95.2)	94.1-96.3	3(0.2)	0.0-0.4	4(0.3)	0.0-0.5	63(4.3)	3.3-5.4	
Burundi 2010	Q1	506(54.7)	51.5-57.9	8(0.9)	0.4-1.7	3(0.3)	0.1-1.0	408(44.1)	40.9-47.3	<0.001
	Q2	566(60.5)	57.3-63.6	5(0.5)	0.2-1.3	1(1.1)	0.0-0.8	364(38.9)	35.8-42.1	
	Q3	549(63.1)	59.8-66.3	9(1.0)	0.5-2.0	7(0.8)	0.4-1.7	305(35.1)	32.0-38.3	
	Q4	571(65.3)	62.4-68.7	9(1.0)	0.5-2.0	3(0.3)	0.1-1.1	287(33.0)	29.9-36.2	

	Q5	984(85.6)	83.5-87.6	15(1.3)	0.8-2.2	1(0.1)	0.0-0.6	149(13.0)	11.1-15.0	
Cambodia 2010	Q1	495(37.8)	35.2-40.5	145(11.1)	9.5-12.9	4(0.3)	0.1-0.8	665(50.8)	48.1-53.5	<0.001
	Q2	451(46.6)	43.6-49.9	145(11.0)	12.9-17.4	0(0.0)	NO	369(38.2)	35.2-41.4	
	Q3	490(59.2)	55.8-62.5	141(17.0)	14.6-19.7	3(0.4)	0.1-1.1	194(23.4)	20.7-26.4	
	Q4	629(73.3)	70.2-76.2	137(16.0)	13.7-18.6	4(0.5)	0.2-1.2	88(10.3)	8.4-12.5	
	Q5	889(88.1)	86.0-90.0	98(9.6)	7.9-11.6	4(0.4)	0.1-1.0	19(1.9)	1.2-2.9	
Cameroon 2011	Q1	268(18.1)	16.2-20.1	29(2.0)	1.4-2.8	3(0.2)	0.1-0.6	1183(79.8)	77.6-81.7	<0.001
	Q2	935(54.9)	52.6-57.3	49(2.9)	2.2-3.8	7(0.4)	0.2-0.9	711(41.8)	39.5-44.1	
	Q3	1164(72.4)	70.2-74.6	60(3.7)	2.9-4.8	3(0.2)	0.1-0.6	380(23.7)	21.6-25.8	
	Q4	1172(86.9)	85.0-88.6	51(3.8)	2.9-4.9	6(0.4)	0.2-1.0	120(8.9)	7.5-10.5	
	Q5	1040(95.3)	93.9-96.4	23(2.1)	1.4-3.2	4(0.4)	0.1-1.0	24(2.2)	1.5-3.2	
CAR 2010	Q1	318(30.7)	28.0-33.6	58(5.6)	4.4-7.2	52(5.0)	3.8-6.5	607(58.7)	55.6-61.6	<0.001
	Q2	416(36.9)	34.1-39.7	77(6.8)	5.5-8.5	80(7.1)	5.7-8.7	555(49.2)	46.3-52.1	
	Q3	520(47.9)	45.0-50.9	64(5.9)	4.6-7.5	103(9.5)	7.9-11.4	398(36.7)	33.9-39.6	
	Q4	506(61.90	58.5-65.1	58(7.1)	5.5-9.1	47(5.80	4.3-7.6	207(25.3)	22.4-28.4	
	Q5	373(77.9)	73.9-81.4	32(6.7)	4.8-9.3	22(4.6)	3.0-6.9	52(10.9)	8.4-14.0	
Chad 2010	Q1	44(4.8)	3.6-6.4	35(3.8)	2.7-5.3	2(0.2)	0.1-0.8	839(91.2)	89.2-92.9	<0.001
	Q2	58(5.6)	4.4-7.2	44(4.3)	3.2-5.7	4(0.4)	0.1-1.0	923(89.7)	87.7-91.4	
	Q3	128(10.2)	8.6-12.0	60(4.8)	3.7-6.1	6(0.5)	0.2-1.1	1062(84.6)	82.4-86.4	
	Q4	282(18.2)	16.3-20.2	145(9.4)	8.0-10.9	13(0.8)	0.5-1.4	1111(71.6)	69.3-73.8	
	Q5	567(40.6)	30.0-43.2	214(15.32)	13.5-17.3	6(0.4)	0.2-1.0	610(43.7)	41.146.3	
Comoros 2012	Q1	304(58.5)	54.2-62.3	39(7.5)	5.5-10.1	0(0.0)	0.0-0.7	177(34.0)	30.1-38.2	<0.001
	Q2	305(74.2)	69.8-78.2	26(6.3)	4.3-9.1	0(0.0)	0.0-0.9	80(19.5)	15.923.6	
	Q3	320(83.8)	79.7-87.1	24(6.3)	4.2-9.2	3(0.8)	0.3-2.4	35(9.2)	6.4-12.5	
	Q4	286(88.3)	84.3-91.4	22(6.8)	4.5-10.1	1(0.3)	0.0-2.2	15(4.6)	2.8-7.5	
	Q5	285(91.1)	87.3-93.8	12(3.8)	2.2-6.3	1(0.3)	0.0-2.2	15(4.8)	2.9-7.8	
	Q1	1914(75.0)	73.3-76.6	140(5.5)	4.7-6.4	7(0.3)	0.1-0.6	492(19.3)	17.8-20.8	<0.001

Congo Brazzaville 2011	Q2	1409(89.5)	87.9-90.9	57(3.6)	2.8-4.7	7(0.4)	0.2-0.9	101(6.4)	5.3-7.7	
	Q3	648(96.1)	94.4-97.4	8(1.2)	0.6-2.4	2(0.3)	0.1-1.2	16(2.4)	1.5-3.8	
	Q4	549(97.0)	95.2-98.1	7(1.2)	0.6-2.6	1(0.2)	0.0-1.2	9(1.6)	0.1-3.0	
	Q5	412(98.6)	96.8-99.4	3(0.7)	0.2-2.2	1(0.2)	0.0-1.7	29(0.5)	0.1-2.0	
Congo D.R. 2013	Q1	1719(56.3)	54.5-58.1	114(3.7)	3.1-4.5	83(2.7)	2.2-3.4	1137(37.2)	35.5-39.0	<0.001
	Q2	1623(64.4)	62.5-66.3	90(3.6)	2.9-4.4	86(3.4)	2.8-4.2	721(28.6)	26.9-30.4	
	Q3	1731(73.6)	71.7-75.3	41(1.7)	1.3-2.4	52(2.2)	1.7-2.9	529(22.5)	20.8-24.2	
	Q4	1709(87.5)	85.9-88.9	34(1.7)	1.2-2.4	22(1.1)	0.1-1.7	189(9.7)	8.4-11.1	
	Q5	1477(97.0)	96.1-97.8	10(0.7)	0.4-1.2	13(0.9)	0.5-1.5	22(1.5)	0.5-2.2	
Costa Rica 2011	Q1	287(95.4)	92.3-97.2	6(2.0)	1.0-4.4	0(0.0)	0.0-1.2	8(2.7)	1.3-5.2	0.009
	Q2	207(99.0)	96.2-99.8	0(0.0)	0.0-1.8	2(1.0)	0.2-3.8	0(0.0)	0.0-1.8	
	Q3	146(99.3)	95.3-99.0	0(0.0)	0.0-2.5	1(0.7)	0.0-4.7	0(0.0)	0.0-2.5	
	Q4	114(99.1)	94.0-99.9	0(0.0)	0.0-3.2	1(0.9)	0.1-6.0	0(0.0)	0.0-3.2	
	Q5	80(97.6)	90.7-99.4	0(0.0)	0.0-4.4	1(1.2)	0.2-8.3	1(1.2)	0.2-8.3	
Cote d'Ivoire 2011	Q1	351(31.9)	29.2-34.8	28(2.6)	1.8-3.7	13(1.2)	0.7-2.0	707(64.3)	61.4-67.1	<0.001
	Q2	533(50.0)	45.0-53.0	19(1.8)	1.1-2.8	12(1.1)	0.6-2.0	502(47.1)	44.1-50.1	
	Q3	570(50.4)	47.5-53.3	18(1.6)	1.0-2.5	6(0.5)	0.2-1.2	537(47.5)	44.6-50.4	
	Q4	617(73.0)	69.9-75.9	33(3.9)	2.8-5.4	5(0.6)	0.2-1.4	190(22.5)	19.8-25.4	
	Q5	587(88.3)	85.5-90.5	12(1.8)	1.0-3.2	2(0.3)	0.1-1.2	64(9.6)	7.6-12.1	
Dominican Republic 2013	Q1	662(93.5)	91.4-95.1	8(1.1)	0.6-2.2	3(0.4)	0.1-1.3	35(4.9)	3.6-6.8	<0.001
	Q2	513(99.2)	97.9-99.7	1(0.2)	0.0-1.4	3(0.6)	0.1-1.5	2(0.4)	0.0-1.4	
	Q3	403(99.0)	97.4-99.6	0(0.0)	0.0-0.9	3(0.7)	0.2-2.3	1(0.3)	0.0-1.7	
	Q4	325(99.1)	97.2-99.7	0(0.0)	0.0-1.1	2(0.6)	0.2-2.4	1(0.3)	0.0-2.1	
	Q5	252(98.8)	96.4-99.6	1(0.4)	0.0-2.7	3(1.2)	0.2-3.1	1(0.4)	0.0-1.4	
Egypt 2008	Q1	742(47.7)	45.2-50.2	151(9.7)	8.3-11.3	3(0.2)	0.1-0.6	659(42.4)	40.0-44.9	<0.001
	Q2	915(64.4)	61.9-66.8	125(8.8)	7.4-10.4	5(0.4)	0.1-0.8	376(26.5)	24.2-28.8	
	Q3	1089(76.7)	74.4-78.8	115(8.1)	6.8-9.6	2(0.1)	0.0-0.6	214(15.1)	13.3-17.0	

	Q4	1086(84.9)	82.8-86.8	82(6.4)	5.2-7.9	5(0.4)	0.2-0.9	106(8.3)	7.0-9.9	
	Q5	1143(95.2)	93.8-96.2	28(2.3)	1.6-3.4	1(0.1)	0.0-0.6	29(2.4)	1.7-3.4	
Ethiopia 2011	Q1	71(3.5)	2.8-4.4	6(0.3)	0.1-0.7	3(0.2)	0.0-0.5	1950(96.1)	95.1-96.9	<0.001
	Q2	43(3.6)	2.7-4.8	6(0.5)	0.2-1.1	3(0.3)	0.1-0.8	1144(95.7)	94.3-96.7	
	Q3	44(4.0)	3.0-5.4	8(0.7)	0.4-1.5	2(0.2)	0.0-0.7	1041(95.1)	93.6-96.2	
	Q4	92(8.5)	7.0-10.3	17(1.6)	1.0-2.5	0(0.0)	0.0-0.3	971(89.9)	88.0-91.6	
	Q5	736(56.6)	53.9-59.3	22(1.7)	1.1-2.6	3(0.2)	0.1-0.7	539(41.5)	38.8-44.2	
Gabon 2012	Q1	1260(69.7)	67.5-71.7	17(0.9)	0.6-1.5	17(0.9)	0.6-1.5	515(28.5)	26.4-30.6	<0.001
	Q2	780(91.0)	88.9-92.8	6(0.7)	0.3-1.6	5(0.6)	0.2-1.4	66(7.7)	6.1-9.7	
	Q3	493(93.6)	91.1-95.4	8(1.5)	0.8-3.0	5(1.0)	0.4-2.3	21(4.0)	2.6-6.0	
	Q4	371(93.7)	90.8-95.7	6(1.5)	0.7-3.3	2(0.5)	0.1-2.0	17(4.3)	2.7-6.8	
	Q5	264(96.4)	93.3-98.0	0(0.0)	0.0-1.3	3(1.1)	0.4-3.3	7(2.6)	1.2-5.3	
Gambia 2013	Q1	547(43.5)	40.8-46.2	14(1.1)	0.7-1.9	67(5.3)	4.2-6.7	630(50.1)	47.3-52.8	<0.001
	Q2	571(42.6)	40.0-45.3	19(1.4)	0.9-2.2	76(5.7)	4.6-7.0	674(50.3)	47.6-53.0	
	Q3	472(43.5)	40.6-46.5	9(0.8)	0.4-1.6	69(6.4)	5.1-8.0	534(49.3)	46.352.2	
	Q4	555(68.4)	65.1-71.5	2(0.3)	0.1-1.0	51(6.3)	4.8-8.2	203(25.0)	22.2-28.1	
	Q5	543(84.2)	81.2-86.8	7(1.1)	0.5-2.3	51(7.9)	6.1-10.3	44(6.8)	5.1-9.0	
Ghana 2011	Q1	493(37.4)	34.8-40.0	14(1.1)	0.1-1.8	8(0.6)	0.3-1.2	805(61.0)	58.3-63.6	<0.001
	Q2	305(52.6)	48.5-56.6	10(1.7)	0.9-3.2	7(1.2)	0.6-2.5	258(44.5)	40.5-48.6	
	Q3	269(68.5)	63.7-72.9	6(1.5)	0.7-3.4	2(0.5)	0.1-2.0	116(29.5)	25.2-34.2	
	Q4	266(82.9)	78.3-86.6	5(1.6)	0.6-3.7	0(0.0)	0.0-1.1	50(15.6)	12.0-20.0	
	Q5	250(96.5)	93.4-98.2	1(0.4)	0.1-2.7	0(0.0)	0.0-1.4	8(3.1)	1.5-6.1	
Guatemala 2008	Q1	448(23.0)	21.2-24.8	5(0.2)	0.1-0.5	4(0.2)	0.1-0.5	1626(76.6)	74.7-78.3	<0.001
	Q2	639(41.5)	39.0-43.9	14(0.9)	0.5-1.5	11(0.7)	0.4-1.3	877(56.9)	54.4-59.4	
	Q3	797(65.3)	62.6-67.9	6(0.5)	0.2-1.1	4(0.3)	0.1-0.9	414(33.9)	31.3-36.6	
	Q4	946(87.6)	85.5-89.4	4(0.4)	0.1-1.0	6(0.6)	0.2-1.2	124(11.5)	9.7-13.5	
	Q5	514(95.36)	93.2-96.8	4(0.7)	0.3-2.0	2(0.4)	0.1-1.5	19(3.5)	2.3-5.5	

Guinea 2012	Q1	179(17.6)	15.3-20.0	19(1.9)	1.2-2.9	10(1.0)	0.5-1.8	812(79.6)	77.0-82.0	<0.001
	Q2	193(1.9)	20.4-26-1	34(4.1)	2.9-5.6	27(3.2)	2.2-4.7	582(69.6)	66.4-72.6	
	Q3	286(33.0)	30.0-36.2	49(5.7)	4.3-7.4	20(2.3)	1.5-3.6	511(59.0)	55.7-62.2	
	Q4	482(52.5)	49.3-55.7	134(14.6)	12.5-17.0	8(0.9)	0.4-1.7	294(32.0)	29.1-35.1	
	Q5	484(81.3)	78.0-84.3	65(10.9)	8.7-13.7	2(0.3)	0.1-1.3	44(7.4)	5.5-9.8	
Guyana 2009	Q1	370(61.6)	57.6-65.4	64(10.7)	8.4-13.4	4(0.7)	0.2-1.8	163(27.1)	23.7-30.8	<0.001
	Q2	211(91.3)	86.9-94.4	6(2.6)	1.2-5.7	2(0.9)	0.2-3.4	12(5.2)	3.0-8.9	
	Q3	228(94.2)	90.5-96.5	4(1.7)	0.6-4.3	6(2.5)	1.1-5.4	4(1.7)	0.6-4.3	
	Q4	172(93.5)	88.8-96.3	7(3.8)	1.8-7.8	0(0.0)	0.0-2.0	5(2.7)	1.1-6.4	
	Q5	139(93.3)	87.9-96.4	4(2.7)	1.0-7.0	0(0.0)	0.0-2.5	6(4.0)	1.8-6.7	
Haiti 2012	Q1	134(10.8)	9.2-12.7	11(1.0)	0.5-1.6	3(0.2)	0.1-0.7	1093(88.1)	86.1-89.8	<0.001
	Q2	219(21.8)	19.3-24.4	12(1.2)	0.7-2.1	6(0.6)	0.3-1.3	769(76.4)	73.7-79.0	
	Q3	440(41.5)	38.6-44.5	14(1.3)	0.8-2.2	6(0.6)	0.3-1.3	600(56.6)	53.6-59.6	
	Q4	408(54.3)	50.7-57.9	28(3.7)	2.6-5.3	2(0.3)	0.1-1.1	313(41.7)	38.2-45.2	
	Q5	381(79.1)	75.2-82.5	12(2.5)	1.4-4.3	0(0.0)	0.0-0.8	89(18.5)	15.2-22.2	
Honduras 2011	Q1	1297(59.1)	57.1-61.2	16(0.7)	0.4-1.2	10(0.5)	0.2-0.8	870(39.7)	37.6-41.7	<0.001
	Q2	1281(81.8)	79.8-83.6	3(0.2)	0.1-0.6	2(0.1)	0.0-0.5	281(17.9)	16.1-19.9	
	Q3	1099(90.2)	88.4-91.8	2(0.2)	0.0-0.7	4(0.3)	0.1-0.9	113(9.3)	7.8-11.0	
	Q4	984(96.9)	95.6-97.8	1(0.1)	0.0-0.7	0(0.0)	0.0-0.4	31(3.1)	2.2-4.3	
	Q5	713(97.7)	96.3-98.5	6(0.8)	0.4-1.8	2(0.3)	0.1-1.1	9(1.2)	0.6-2.4	
India 2005	Q1	703(13.2)	12.3-14.1	395(7.4)	6.7-8.2	11(0.2)	0.1-0.4	4215(79.2)	78.1-80.2	<0.001
	Q2	1350(23.9)	22.8-25.1	488(8.7)	7.9-9.4	13(0.2)	0.1-0.4	3787(67.2)	65.9-68.4	
	Q3	2542(40.7)	39.5-42.0	621(10.0)	9.2-10.7	26(0.4)	0.3-0.6	3052(48.9)	47.7-50.1	
	Q4	4086(60.6)	59.4-61.7	633(9.4)	8.7-10.1	18(0.3)	0.2-0.4	2010(29.8)	28.7-30.9	
	Q5	5496(85.2)	84.3-86.0	308(4.8)	4.3-5.3	21(0.3)	0.2-0.5	627(9.7)	9.0-10.5	
Indonesia 2012	Q1	866(26.4)	24.9-27.9	960(29.2)	27.7-30.8	6(0.2)	0.1-0.4	1453(44.2)	42.5-45.9	<0.001
	Q2	1223(55.0)	52.9-57.0	625(28.1)	26.3-30.0	5(0.2)	0.1-0.5	372(16.7)	15.2-18.3	

	Q3	1326(67.0)	64.9-69.1	478(24.2)	22.3-26.1	2(0.1)	0.0-0.4	172(8.7)	7.5-10.0	
	Q4	1473(79.8)	77.9-81.6	303(16.4)	14.8-18.2	1(0.1)	0.0-0.4	69(3.7)	3.0-4.7	
	Q5	1388(88.0)	86.3-89.5	152(9.6)	8.3-11.2	4(0.3)	0.1-0.7	34(2.2)	1.5-3.0	
Iraq 2011	Q1	2902(63.3)	61.9-64.6	652(14.2)	13.2-15.3	11(0.2)	0.1-0.4	1022(22.3)	21.1-23.5	<0.001
	Q2	2411(73.9)	72.4-75.4	473(14.5)	13.3-15.7	26(0.8)	0.5-1.2	353(10.8)	9.8-11.9	
	Q3	2063(78.1)	76.5-79.7	378(14.3)	13.0-15.7	21(0.8)	0.5-1.2	179(6.8)	5.9-7.8	
	Q4	1641(81.2)	79.5-82.9	258(12.8)	11.4-14.3	8(0.4)	0.2-0.8	113(5.6)	4.7-6.7	
	Q5	1244(83.9)	81.9-85.7	177(11.9)	10.4-13.7	5(0.3)	0.1-0.8	57(3.8)	3.0-5.0	
Jamaica 2011	Q1	148(94.9)	90.0-97.4	4(2.6)	1.0-6.7	0(0.0)	0.0-2.3	4(2.6)	0.1-6.7	0.015
	Q2	139(99.3)	95.1-99.9	0(0.0)	0.0-2.6	1(0.7)	0.1-4.9	0(0.0)	0.0-2.6	
	Q3	133(100.0)	97.3-100.0	0(0.0)	0.0-2.7	0(0.0)	0.0-2.7	0(0.0)	0.0-2.7	
	Q4	105(99.1)	93.3-99.9	1(0.9)	0.1-6.5	0(0.0)	0.0-3.4	0(0.0)	0.0-3.4	
	Q5	95(100.0)	96.2-100.0	0(0.0)	0.0-3.8	0(0.0)	0.0-3.8	0(0.0)	0.0-3.8	
Jordan 2012	Q1	1601(98.2)	87.4-98.7	19(1.2)	0.7-1.8	1(0.1)	0.0-0.4	10(0.6)	0.3-1.1	<0.001
	Q2	1648(99.6)	99.2-99.8	5(0.3)	0.1-0.7	0(0.0)	0.0-0.2	1(0.1)	0.0-0.4	
	Q3	1386(99.6)	99.0-99.8	6(0.4)	0.2-1.0	0(0.0)	0.0-0.3	0(0.0)	0.0-0.3	
	Q4	993(99.5)	98.8-99.8	1(0.1)	0.0-0.7	0(0.0)	0.0-0.4	4(0.4)	0.2-1.1	
	Q5	442(100.0)	99.2-100.0	0(0.0)	0.0-0.8	0(0.0)	0.0-0.8	0(0.0)	0.0-0.8	
Kazakhstan 2010	Q1	380(98.7)	96.9-99.5	3(0.8)	0.3-2.4	0(0.0)	0.0-1.0	2(0.5)	0.1-2.1	0.066
	Q2	423(99.8)	98.3-100.0	1(0.2)	0.0-1.7	0(0.0)	0.0-0.9	0(0.0)	0.0-0.9	
	Q3	429(99.8)	98.4-100.0	0(0.0)	0.0-0.9	0(0.0)	0.0-0.9	1(0.2)	0.0-1.6	
	Q4	374(100.0)	99.0-100.0	0(0.0)	0.0-1.0	0(0.0)	0.0-1.0	0(0.0)	0.0-1.0	
	Q5	414(100.0)	99.1-100.0	0(0.0)	0.0-0.9	0(0.0)	0.0-0.9	0(0.0)	0.0-0.9	
Kenya 2008	Q1	184(17.2)	15.0-19.5	49(4.6)	3.5-6.0	0(0.0)	0.0-0.3	839(78.3)	75.7-80.6	<0.001
	Q2	210(31.3)	27.9-34.9	12(1.8)	1.0-3.1	1(0.2)	0.0-1.1	448(66.8)	63.1-70.2	
	Q3	270(43.9)	40.0-47.9	6(1.0)	0.4-2.2	1(0.2)	0.0-1.1	338(55.0)	51.0-58.9	
	Q4	333(56.0)	51.9-59.9	14(2.4)	1.4-3.9	2(0.3)	0.1-1.3	246(41.3)	37.4-45.4	

	Q5	633(81.2)	78.3-83.7	6(0.8)	0.3-1.7	2(0.3)	0.1-1.0	139(17.8)	15.3-20.7	
Kyrgyzstan 2012	Q1	597(98.8)	97.6-99.4	3(0.5)	0.2-1.5	1(0.2)	0.0-1.2	3(0.5)	0.2-1.5	0.308
	Q2	606(99.5)	98.5-99.4	2(0.3)	0.1-1.3	0(0.0)	0.0-0.6	1(0.2)	0.0-1.2	
	Q3	617(98.6)	97.3-99.3	3(0.5)	0.2-1.5	0(0.0)	0.0-0.6	6(1.0)	0.4-2.1	
	Q4	528(98.5)	97.0-99.3	1(0.2)	0.0-1.3	1(0.2)	0.0-1.3	6(1.1)	0.5-2.5	
	Q5	386(100.0)	99.1-100.0	0(0.0)	0.0-1.0	0(0.0)	0.0-1.0	0(0.0)	0.0-1.0	
Lao 2011	Q1	110(8.1)	6.7-9.6	17(1.2)	0.8-2.0	18(1.3)	0.8-2.1	1222(89.4)	87.6-90.9	<0.001
	Q2	213(21.1)	18.7-23.7	30(3.0)	2.1-4.2	3(0.3)	0.1-0.9	764(75.6)	72.9-78.2	
	Q3	325(38.3)	35.1-41.6	57(6.7)	5.2-8.6	2(0.2)	0.1-0.9	464(54.7)	51.3-58.0	
	Q4	368(55.1)	51.3-58.8	57(8.5)	6.6-10.9	0(0.0)	0.0-0.5	243(36.4)	32.8-40.1	
	Q5	480(87.1)	84.0-89.7	21(3.8)	2.5-5.8	1(0.2)	0.0-1.3	49(9.0)	6.8-11.6	
Lesotho 2009	Q1	277(36.9)	33.5-40.5	13(1.7)	1.0-3.0	1(0.1)	0.0-0.9	459(61.2)	57.7-64.6	<0.001
	Q2	282(49.8)	45.7-53.9	18(3.2)	2.0-5.0	1(0.2)	0.0-1.2	265(46.8)	42.7-50.9	
	Q3	263(55.3)	50.7-59.7	23(4.8)	3.2-7.2	3(0.6)	0.2-1.9	187(39.3)	35.0-48.3	
	Q4	298(73.8)	69.2-77.8	13(3.2)	1.9-5.5	2(0.5)	0.1-2.0	91(22.5)	18.7-26.9	
	Q5	280(89.2)	85.2-92.2	10(3.2)	1.7-5.8	2(0.6)	0.2-2.5	22(7.0)	4.7-10.4	
Liberia 2013	Q1	696(42.5)	40.1-44.9	58(3.5)	2.7-4.6	32(2.0)	1.4-2.7	853(52.0)	49.6-54.5	<0.001
	Q2	637(51.3)	48.5-54.1	67(5.4)	4.3-6.8	29(2.3)	1.6-3.3	509(41.0)	38.3-43.7	
	Q3	573(61.2)	58.0-64.2	53(5.7)	4.3-7.3	23(2.5)	1.6-3.7	288(30.7)	27.9-33.8	
	Q4	353(70.2)	66.0-74.0	40(8.0)	5.9-10.7	6(1.2)	0.5-2.6	104(20.7)	17.4-24.4	
	Q5	224(81.8)	76.7-85.9	18(6.6)	4.2-10.2	0(0.0)	0.0-1.3	32(11.68)	8.4-16.1	
Macedonia 2011	Q1	89(95.7)	89.0-98.4	2(2.2)	0.5-8.3	0(0.0)	0.0-3.9	2(2.2)	0.5-8.3	0.198
	Q2	96(97.00	91.0-99.0	0(0.0)	0.0-3.7	1(1.0)	0.1-6.9	2(2.0)	0.5-7.8	
	Q3	106(99.1)	93.6-99.9	1(0.9)	0.1-6.4	0(0.0)	0.0-3.4	0(0.0)	0.0-3.4	
	Q4	104(100.0)	96.5-100.0	0(0.0)	0.0-3.5	0(0.0)	0.0-3.5	0(0.0)	0.0-3.5	
	Q5	97(97.0)	91.0-99.0	1(1.0)	0.1-6.8	2(2.0)	0.5-7.7	0(0.0)	0.0-3.6	
	Q1	407(18.9)	17.3-20.6	92(4.3)	3.5-5.2	3(0.1)	0.0-0.4	1657(76.8)	74.9-78.5	<0.001

Madagascar 2008	Q2	407(25.8)	23.7-28.0	74(4.7)	3.8-5.9	2(0.1)	0.0-0.5	1094(69.4)	67.1-71.6	
	Q3	486(36.6)	34.0-39.2	89(6.7)	5.5-8.2	6(0.5)	0.2-1.0	748(56.3)	53.6-58.9	
	Q4	561(47.1)	44.3-49.9	162(13.6)	11.8-15.7	1(0.1)	0.0-0.6	467(39.2)	36.5-42.0	
	Q5	778(67.1)	64.4-69.8	262(22.6)	20.3-25.1	4(0.4)	0.1-0.9	115(9.9)	8.3-11.8	
Malawi 2010	Q1	1799(64.6)	62.8-66.4	13(0.5)	0.3-0.8	45(1.6)	1.2-2.2	927(33.3)	31.6-35.1	<0.001
	Q2	1886(67.7)	66.0-69.5	6(0.2)	0.1-0.5	67(2.4)	1.9-3.0	825(29.6)	28.0-31.4	
	Q3	1933(70.6)	68.9-72.3	12(0.4)	0.2-0.8	73(2.7)	2.1-3.3	719(26.3)	24.7-28.0	
	Q4	1751(78.2)	76.5-79.9	9(0.4)	0.2-0.8	59(2.6)	2.0-3.4	419(18.7)	17.2-20.4	
	Q5	1415(88.8)	87.2-90.3	8(0.5)	0.3-1.0	29(1.8)	1.3-2.6	141(8.9)	7.6-10.3	
Maldives 2009	Q1	484(92.0)	89.4-94.0	4(0.8)	0.3-2.0	14(2.7)	1.6-4.4	24(4.6)	3.1-6.7	<0.001
	Q2	650(94.5)	92.5-96.0	4(0.6)	0.2-1.5	11(1.6)	0.9-2.9	23(3.3)	2.2-5.0	
	Q3	627(96.3)	94.6-97.5	5(0.8)	0.3-1.8	6(0.9)	0.4-2.0	13(2.0)	1.2-3.4	
	Q4	366(97.9)	95.8-98.9	4(1.1)	0.4-2.8	1(0.3)	0.0-1.9	3(0.8)	0.3-2.5	
	Q5	198(97.1)	93.6-98.7	6(2.9)	1.3-6.4	0(0.0)	0.0-1.8	0(0.0)	0.0-1.8	
Mali 2012	Q1	330(28.7)	26.2-31.4	87(7.6)	6.2-9.2	12(1.0)	0.6-1.8	721(62.7)	59.9-65.4	<0.001
	Q2	475(39.6)	36.9-42.4	58(4.8)	3.8-6.2	15(1.3)	0.8-2.1	651(54.3)	51.5-57.1	
	Q3	571(47.8)	45.0-50.7	41(3.4)	2.5-4.6	16(1.3)	0.8-2.2	566(47.4)	44.6-50.2	
	Q4	873(74.2)	71.7-76.7	40(3.4)	2.5-4.6	7(0.6)	0.3-1.2	256(21.8)	19.5-24.2	
	Q5	1222(94.2)	92.8-95.4	15(1.2)	0.7-1.9	10(0.8)	0.4-1.4	50(3.9)	2.9-5.1	
Moldova 2012	Q1	82(97.6)	90.9-99.4	0(0.0)	0.0-4.3	0(0.0)	0.0-4.3	2(2.4)	0.6-9.1	0.081
	Q2	117(100.0)	96.9-100.0	0(0.0)	0.0-3.1	0(0.0)	0.0-3.1	0(0.0)	0.0-3.1	
	Q3	115(100.0)	96.8-100.0	0(0.0)	0.0-3.2	0(0.0)	0.0-3.2	0(0.0)	0.0-3.2	
	Q4	130(97.7)	93.2-99.3	2(1.5)	0.4-5.9	0(0.0)	0.0-2.7	1(0.8)	0.1-5.2	
	Q5	272(99.3)	97.1-99.8	0(0.0)	0.0-1.3	0(0.0)	0.0-1.3	2(0.7)	0.2-2.9	
Mongolia 2010	Q1	481(97.0)	95.0-98.2	3(0.6)	0.2-1.9	0(0.0)	0.0-0.7	12(2.4)	1.4-4.2	0.069
	Q2	382(98.5)	96.6-99.3	3(0.8)	0.2-2.4	1(0.3)	0.0-1.8	2(0.5)	0.1-2.0	
	Q3	316(98.8)	96.7-99.5	0(0.0)	0.0-1.2	1(0.3)	0.0-2.2	3(0.9)	0.3-2.9	

	Q4	250(99.6)	97.2-99.9	0(0.0)	0.0-1.5	1(0.4)	0.1-2.8	0(0.0)	0.0-1.5	
	Q5	233(99.2)	96.6-99.8	1(0.4)	0.1-3.0	0(0.0)	0.0-1.6	1(0.4)	0.1-3.0	
Montenegro 2013	Q1	79(98.8)	91.5-99.8	0(0.0)	0.0-4.5	0(0.0)	0.0-4.5	1(1.3)	0.2-8.5	0.686
	Q2	103(100.0)	96.4-100.0	0(0.0)	0.0-3.5	0(0.0)	0.0-3.5	0(0.0)	0.0-3.5	
	Q3	100(99.0)	93.2-99.9	0(0.0)	0.0-3.6	0(0.0)	0.0-3.6	1(1.0)	0.1-6.8	
	Q4	104(99.1)	93.5-99.9	0(0.0)	0.0-3.5	0(0.0)	0.0-3.5	1(1.0)	0.1-6.5	
	Q5	105(100.0)	96.6-100.0	0(0.0)	0.0-3.5	0(0.0)	0.0-3.5	0(0.0)	0.0-3.5	
Mozambique 2011	Q1	422(34.7)	31.1-37.4	25(2.1)	1.4-3.0	27(2.2)	1.5-3.2	742(61.0)	58.2-63.7	<0.001
	Q2	560(42.1)	39.5-44.8	16(1.2)	0.7-2.0	25(1.9)	1.3-2.8	729(54.8)	52.1-57.5	
	Q3	751(54.4)	51.7-57.0	26(1.9)	1.3-2.8	46(3.3)	2.5-4.4	558(40.4)	37.8-43.6	
	Q4	1119(74.1)	71.8-76.2	23(1.5)	1.0-2.3	42(2.8)	2.1-3.7	327(21.6)	19.6-23.8	
	Q5	1349(90.0)	88.4-91.4	6(0.4)	0.2-0.9	42(2.8)	2.1-3.8	102(6.8)	5.6-8.2	
Namibia 2013	Q1	485(70.6)	67.1-73.9	14(2.0)	1.2-3.4	2(0.3)	0.1-1.2	186(27.1)	23.9-30.5	<0.001
	Q2	588(83.8)	80.8-86.3	17(2.4)	1.5-3.9	1(0.1)	0.0-1.0	96(13.7)	11.3-16.4	
	Q3	611(86.4)	83.7-88.8	10(1.4)	1.8-2.6	4(0.6)	0.2-1.5	82(11.6)	9.4-14.2	
	Q4	624(94.8)	92.9-96.3	4(0.6)	0.2-1.6	4(0.6)	0.2-1.6	26(4.0)	2.7-5.7	
	Q5	397(97.5)	95.5-98.7	3(0.7)	0.2-2.3	0(0.0)	0.0-0.9	7(1.7)	0.8-3.6	
Nepal 2011	Q1	137(14.5)	12.4-16.9	4(0.4)	0.2-1.1	15(1.6)	1.0-2.6	788(83.5)	81.0-85.7	<0.001
	Q2	180(28.5)	25.1-32.1	4(0.6)	0.2-1.7	11(1.7)	1.0-3.1	437(69.2)	65.4-72.6	
	Q3	250(44.7)	40.6-48.9	5(0.9)	0.4-2.1	13(2.3)	1.4-4.0	291(52.1)	47.9-56.2	
	Q4	305(58.1)	53.8-62.3	18(3.4)	2.2-5.4	8(1.5)	0.8-3.0	194(37.0)	32.9-41.2	
	Q5	378(85.5)	81.9-88.5	9(2.0)	1.1-3.9	1(0.2)	0.0-1.6	54(12.2)	9.5-15.6	
Niger 2012	Q1	177(13.4)	11.6-15.3	5(0.4)	0.2-0.9	23(1.7)	1.2-2.6	1121(84.5)	82.5-86.4	<0.001
	Q2	276(20.9)	18.8-23.2	7(0.5)	0.3-1.1	23(1.7)	1.2-2.6	1016(76.9)	94.5-99.0	
	Q3	325(23.2)	21.0-25.4	10(0.7)	0.4-1.3	18(1.3)	0.8-2.0	1050(74.8)	72.5-77.0	
	Q4	516(34.2)	31.9-36.7	15(1.0)	0.6-1.6	17(1.1)	0.7-1.8	960(63.7)	61.2-66.1	
	Q5	1539(78.7)	76.8-80.4	19(0.5)	0.6-1.5	9(0.5)	0.2-0.9	389(19.9)	18.2-21.7	

Nigeria 2013	Q1	249(6.1)	5.4-6.8	30(0.7)	0.5-1.0	45(1.1)	0.8-1.5	3777(92.1)	91.2-92.9	<0.001
	Q2	741(16.8)	15.7-17.9	89(2.0)	1.6-2.5	131(3.0)	2.5-3.5	3462(78.3)	77.0-79.5	
	Q3	1376(36.5)	34.9-38.0	172(4.6)	3.9-5.3	103(2.7)	2.3-3.3	2122(56.2)	54.7-57.8	
	Q4	1977(56.4)	54.8-58.1	228(6.5)	5.7-7.4	61(1.7)	1.4-2.2	1237(35.3)	33.7-36.9	
	Q5	2470(80.5)	79.0-81.8	179(5.8)	5.1-6.7	26(0.9)	0.6-1.2	394(12.8)	11.7-14.1	
Pakistan 2012	Q1	412(26.6)	24.5-28.9	48(3.1)	2.3-4.1	7(0.5)	0.2-0.9	1082(69.9)	67.5-72.1	<0.001
	Q2	582(42.2)	39.6-44.8	67(4.9)	3.8-6.1	7(0.5)	0.2-1.1	724(52.5)	49.8-55.1	
	Q3	684(51.3)	48.6-54.0	71(5.3)	4.2-6.7	8(0.6)	0.3-1.2	570(42.8)	40.1-45.4	
	Q4	856(66.5)	63.8-69.0	65(5.1)	4.0-6.4	6(0.5)	0.2-1.0	361(28.0)	25.6-30.5	
	Q5	1103(84.9)	82.9-86.8	30(2.3)	1.6-3.3	8(0.6)	0.3-1.2	158(12.2)	10.5-14.1	
Peru 2012	Q1	967(58.8)	56.4-61.2	40(4.9)	3.9-6.0	24(1.5)	1.0-2.2	573(34.9)	32.6-37.2	<0.001
	Q2	1369(86.8)	85.0-88.4	38(2.4)	1.8-3.3	12(0.8)	0.4-1.3	158(10.0)	8.6-11.6	
	Q3	1140(95.7)	94.4-96.7	15(1.3)	0.8-2.1	0(0.0)	0.0-0.3	36(3.0)	2.2-4.2	
	Q4	777(98.7)	97.7-99.3	5(0.6)	0.3-1.5	1(0.1)	0.0-0.9	4(0.5)	0.2-1.3	
	Q5	504(99.0)	97.7-99.6	4(0.8)	0.3-2.1	0(0.0)	0.0-0.7	1(0.2)	0.0-1.4	
Philippines 2013	Q1	481(35.9)	33.3-38.5	102(7.6)	6.3-9.2	1(0.1)	0.0-0.5	757(56.5)	53.8-59.1	<0.001
	Q2	550(58.8)	55.6-61.9	119(12.7)	10.7-15.0	1(0.1)	0.0-0.8	265(28.3)	25.5-31.3	
	Q3	612(73.3)	70.2-76.2	96(11.5)	9.5-13.8	2(0.2)	0.1-1.0	125(15.0)	12.7-17.6	
	Q4	583(86.9)	84.1-89.2	49(7.3)	5.6-9.5	3(0.5)	0.1-1.4	36(5.4)	3.9-7.4	
	Q5	443(93.3)	90.6-95.2	18(3.8)	2.4-5.9	0(0.0)	0.0-0.8	14(3.0)	1.8-4.9	
Rwanda 2010	Q1	858(69.6)	67.0-72.1	4(0.3)	0.1-0.9	5(0.4)	0.2-1.0	365(29.6)	27.1-32-2	<0.001
	Q2	809(71.5)	68.8-74.1	4(0.4)	0.1-0.9	2(0.2)	0.0-0.7	316(27.94)	25.4-30.6	
	Q3	757(75.0)	72.2-77.5	3(0.3)	0.1-0.9	4(0.2)	0.1-1.1	246(24.4)	21.8-27.1	
	Q4	750(80.7)	78.1-83.1	3(0.3)	0.1-1.0	2(0.2)	0.1-0.9	174(18.7)	16.3-21.4	
	Q5	812(90.5)	88.4-92.3	5(0.6)	0.2-1.3	4(0.5)	0.2-1.2	76(8.5)	6.8-10.5	
	Q1	201(70.8)	65.2-75.8	15(5.3)	3.2-8.6	0(0.0)	0.0-1.3	68(23.9)	19.3-29.3	<0.001
	Q2	215(72.9)	67.5-77.7	11(3.7)	2.1-6.6	1(0.3)	0.0-2.4	68(23.1)	18.6-28.2	

São Tome & Principe 2008	Q3	200(79.7)	74.2-84.2	6(2.4)	1.1-5.2	0(0.0)	0.0-1.5	45(17.9)	13.6-23.2	
	Q4	190(86.4)	81.1-90.3	5(2.3)	0.9-5.4	2(0.9)	0.2-3.6	23(10.5)	7.0-15.3	
	Q5	133(88.1)	81.8-92.4	7(4.6)	2.2-9.4	1(0.7)	0.1-4.6	10(6.6)	3.6-11.9	
Senegal 2014	Q1	421(30.1)	27.7-32.6	12(0.9)	0.5-1.5	239(17.1)	15.2-19.1	727(52.0)	49.3-54.6	<0.001
	Q2	497(44.8)	41.9-47.7	7(0.6)	0.3-1.3	265(23.9)	21.5-26.5	341(30.7)	28.1-33.5	
	Q3	533(64.7)	61.4-67.9	7(0.9)	0.4-1.8	194(23.5)	20.8-26.6	90(10.9)	9.0-13.2	
	Q4	377(79.2)	75.3-82.6	5(1.1)	0.4-2.5	66(13.9)	11.0-17.3	28(5.9)	4.1-8.4	
	Q5	263(80.7)	76.0-84.6	7(2.2)	1.0-4.4	48(14.7)	11.3-19.0	8(2.5)	1.2-4.8	
Serbia 2014	Q1	133(97.1)	92.4-98.9	0(0.0)	0.0-2.7	0(0.0)	0.0-2.7	4(2.9)	1.1-7.6	0.106
	Q2	159(100.0)	97.7-100.0	0(0.0)	0.0-2.3	0(0.0)	0.0-2.3	0(0.0)	0.0-2.3	
	Q3	196(99.5)	96.5-99.9	1(0.5)	0.1-3.5	0(0.0)	0.0-1.9	0(0.0)	0.0-1.9	
	Q4	209(99.5)	96.7-99.9	0(0.0)	0.0-1.7	0(0.0)	0.0-1.7	1(0.5)	0.1-3.3	
	Q5	252(98.4)	95.9-99.4	1(0.4)	0.1-2.7	0(0.0)	0.0-1.4	3(1.2)	0.4-3.6	
Sierra Leone 2013	Q1	814(49.6)	47.2-52.0	62(3.8)	3.0-4.8	17(1.0)	0.6-1.7	748(45.6)	43.2-48.0	<0.001
	Q2	737(52.1)	49.4-54.6	48(3.4)	2.6-4.5	14(1.0)	0.6-1.7	617(43.6)	41.0-46.2	
	Q3	774(53.2)	50.6-55.7	73(5.0)	4.0-6.3	9(0.6)	0.3-1.2	600(41.2)	38.7-43.8	
	Q4	1019(66.0)	63.6-68.3	125(8.1)	6.8-9.6	11(0.7)	0.4-1.3	390(25.2)	23.1-27.5	
	Q5	786(75.8)	73.1-78.3	112(10.8)	9.1-12.8	7(0.7)	0.3-1.4	132(12.7)	10.8-14.9	
St Lucia 2012	Q1	20(95.24)	71.3-79.4	0(0.0)	0.0-16.1	1(4.76)	0.6-28.7	0(0.0)	0.0-16.1	0.447
	Q2	26(100.0)	86.8-100.0	0(0.0)	0.0-13.2	0(0.0)	0.0-13.2	0(0.0)	0.0-13.2	
	Q3	22(100.0)	84.6-100.0	0(0.0)	0.0-15.4	0(0.0)	0.0-15.4	0(0.0)	0.0-15.4	
	Q4	17(100.0)	80.5-100.0	0(0.0)	0.0-19.5	0(0.0)	0.0-19.5	0(0.0)	0.0-19.5	
	Q5	12(100.0)	73.5-100.0	0(0.0)	0.0-26.5	0(0.0)	0.0-26.5	0(0.0)	0.0-26.5	
State of Palestine 2010	Q1	965(97.7)	96.5-98.4	11(1.1)	0.6-2.0	4(0.4)	0.2-1.1	8(0.8)	0.4-1.6	0.086
	Q2	1020(98.0)	96.9-98.7	18(1.7)	1.1-2.7	0(0.0)	0.0-0.4	3(0.3)	0.1-0.9	
	Q3	896(98.3)	98.0-99.4	5(0.6)	0.2-1.3	1(0.1)	0.0-0.8	4(0.4)	0.2-1.2	
	Q4	810(98.3)	97.2-99.0	11(1.3)	0.7-2.4	1(0.1)	0.0-0.9	2(0.2)	0.1-1.0	

	Q5	562(98.4)	97.0-99.2	7(1.2)	0.6-2.6	2(0.4)	0.1-1.4	0(0.0)	0.0-0.6	
Suriname 2010	Q1	473(73.3)	69.8-76.6	49(7.6)	5.8-9.9	75(11.6)	9.4-14.3	48(7.4)	5.7-9.7	<0.001
	Q2	194(90.20	85.5-93.6	8(3.7)	1.9-7.3	7(3.3)	1.6-6.7	6(2.8)	1.3-6.1	
	Q3	159(94.1)	89.3-96.8	7(4.1)	2.0-8.5	2(1.2)	0.3-4.6	1(0.6)	0.1-4.1	
	Q4	127(96.2)	81.2-98.4	3(2.3)	0.7-6.8	1(0.8)	0.1-5.2	1(0.8)	0.1-5.2	
	Q5	100(96.2)	90.1-98.6	1(1.0)	0.1-6.6	2(2.0)	0.5-7.4	1(1.0)	0.1-6.6	
Swaziland 2010	Q1	125(62.3)	55.3-68.6	2(1.0)	0.2-3.9	3(1.5)	0.5-4.5	71(35.3)	29.0-42.2	<0.001
	Q2	141(74.2)	67.5-80.0	4(2.1)	0.8-5.5	4(2.1)	0.8-5.5	41(21.5)	16.3-28.0	
	Q3	185(84.5)	79.0-88.7	4(1.8)	0.7-4.8	1(0.5)	0.1-3.2	29(13.2)	9.3-18.4	
	Q4	173(85.2)	79.6-89.5	7(3.5)	1.6-7.1	3(1.5)	0.5-4.5	20(9.9)	6.4-14.8	
	Q5	188(91.7)	87.0-94.8	4(2.0)	0.7-5.1	1(0.5)	0.1-3.4	12(5.9)	3.3-10.0	
Tajikistan 2012	Q1	325(58.9)	54.7-62.9	91(16.5)	13.6-19.8	3(0.5)	0.2-1.7	133(24.1)	20.7-27.8	<0.001
	Q2	424(69.2)	65.4-72.7	104(17.0)	14.2-20.2	2(0.3)	0.1-1.3	83(13.5)	11.0-16.5	
	Q3	503(78.7)	75.4-81.7	84(13.2)	10.7-16.0	2(0.3)	0.1-1.2	50(7.8)	6.0-10.0	
	Q4	557(88.6)	85.8-90.8	33(5.3)	3.8-7.3	4(0.6)	0.2-1.7	35(5.6)	4.0-7.7	
	Q5	697(91.5)	89.3-93.3	40(5.3)	3.9-7.1	2(0.3)	0.1-1.0	23(3.0)	2.0-4.5	
Tanzania 2010	Q1	292(30.3)	27.5-33.3	5(0.5)	0.2-1.2	8(0.8)	0.4-1.7	658(68.3)	65.3-71.2	<0.001
	Q2	396(35.9)	33.1-38.8	13(1.2)	0.7-2.0	14(1.3)	0.8-2.1	680(61.7)	58.7-64.5	
	Q3	459(44.7)	41.7-47.8	17(1.7)	1.0-2.7	6(0.6)	0.3-1.3	544(53.0)	50.0-56.1	
	Q4	567(57.3)	54.2-60.4	20(2.0)	1.3-3.1	2(0.2)	0.1-0.8	400(40.4)	37.4-43.5	
	Q5	612(81.4)	78.4-84.0	26(3.5)	2.4-5.0	4(0.5)	0.2-1.4	110(14.6)	12.3-17.4	
Timor Leste 2009	Q1	94(6.7)	5.5-8.1	79(5.6)	4.5-6.9	2(0.1)	0.0-0.6	1238(87.6)	85.8-89.2	<0.001
	Q2	104(8.7)	7.2-10.4	85(7.1)	5.8-8.7	2(0.2)	0.0-0.7	1011(84.1)	81.9-86.1	
	Q3	204(16.0)	14.1-18.1	119(9.3)	7.8-11.0	2(0.2)	0.0-0.6	953(74.6)	72.1-76.9	
	Q4	340(29.6)	27.0-32.3	119(10.4)	8.7-12.3	2(0.2)	0.0-0.7	687(59.8)	57.0-62.6	
	Q5	503(58.2)	54.8-61.4	97(11.2)	9.3-13.5	1(0.1)	0.0-0.8	264(30.5)	27.5-33.7	
	Q1	341(27.5)	25.1-30.1	1(0.1)	0.0-0.6	217(17.5)	15.5-19.7	681(54.9)	52.1-57.7	<0.001

Togo 2013	Q2	332(38.3)	35.2-41.6	2(0.2)	0.1-0.9	198(22.9)	20.2-25.8	334(38.6)	35.4-41.9	
	Q3	457(55.2)	51.8-58.6	1(0.1)	0.0-0.9	198(23.9)	21.1-26.9	172(20.8)	18.1-23.7	
	Q4	590(88.2)	55.5-90.4	3(0.5)	0.1-1.4	46(6.9)	5.2-9.1	30(4.5)	3.2-6.3	
	Q5	609(95.1)	93.0-96.4	5(0.8)	0.3-1.9	15(2.3)	1.4-3.8	12(1.9)	1.1-3.3	
Tunisia 2011	Q1	276(91.4)	87.6-94.1	0(0.0)	0.0-1.2	0(0.0)	0.0-1.2	26(8.6)	5.9-12.4	<0.001
	Q2	248(99.2)	96.8-99.8	1(0.4)	0.1-2.8	0(0.0)	0.0-1.5	1(0.4)	0.1-2.8	
	Q3	195(99.5)	96.4-99.9	1(0.5)	0.1-3.6	0(0.0)	0.0-1.9	0(0.0)	0.0-1.9	
	Q4	233(99.6)	97.0-99.9	0(0.0)	0.0-1.6	0(0.0)	0.0-1.6	1(0.4)	0.1-3.0	
	Q5	152(99.4)	95.5-99.9	0(0.0)	0.0-2.4	0(0.0)	0.0-2.4	1(0.7)	0.1-4.5	
Uganda 2011	Q1	464(38.2)	35.5-41.0	26(2.1)	1.5-3.1	14(1.2)	0.7-1.9	711(58.5)	55.7-61.3	<0.001
	Q2	499(53.4)	50.2-56.6	12(1.3)	0.7-2.2	13(1.4)	0.8-2.4	411(44.0)	40.8-47.2	
	Q3	493(57.6)	54.3-60.9	12(1.4)	0.8-2.5	16(1.9)	1.4-3.0	335(39.1)	35.9-42.5	
	Q4	471(62.3)	58.8-65.7	21(2.8)	1.8-4.2	11(1.5)	0.8-2.6	253(33.5)	30.2-36.9	
	Q5	894(89.5)	87.4-91.2	13(1.3)	0.8-2.2	9(0.9)	0.5-1.7	83(8.3)	6.7-10.2	
Ukraine 2012	Q1	324(98.8)	96.8-99.5	2(0.6)	0.2-2.4	0(0.0)	0.0-1.1	2(0.6)	0.2-2.4	0.05
	Q2	369(97.9)	95.8-98.9	0(0.0)	0.0-1.0	0(0.0)	0.0-1.0	8(2.1)	1.1-4.2	
	Q3	249(99.2)	96.9-99.8	0(0.0)	0.0-1.5	0(0.0)	0.0-1.5	2(0.8)	0.2-3.1	
	Q4	300(100.0)	98.8-100.0	0(0.0)	0.0-1.2	0(0.0)	0.0-1.2	0(0.0)	0.0-1.2	
	Q5	304(98.7)	96.6-99.5	2(0.7)	0.2-2.6	0(0.0)	0.0-1.2	2(0.7)	0.2-2.6	
Vietnam 2010	Q1	200(61.2)	55.8-66.3	7(2.1)	1.0-4.4	3(0.9)	0.3-2.8	117(35.8)	30.8-41.1	<0.001
	Q2	210(94.2)	90.2-96.6	3(1.4)	0.4-4.1	2(0.9)	0.2-3.5	8(3.6)	1.8-7.0	
	Q3	237(98.8)	96.2-99.6	1(0.4)	0.1-2.9	0(0.0)	0.0-1.5	2(0.8)	1.2-3.3	
	Q4	263(98.1)	95.6-99.2	3(1.1)	0.4-3.4	0(0.0)	0.0-1.4	2(0.8)	0.2-2.9	
	Q5	304(99.7)	97.7-100.0	0(0.0)	0.0-1.2	0(0.0)	0.0-1.2	1(0.3)	0.0-2.3	
Zambia 2007	Q1	237(27.6)	24.7-30.6	1(0.1)	0.0-0.8	15(1.7)	1.1-2.9	607(70.6)	67.4-73.5	<0.001
	Q2	237(27.5)	24.6-30.6	6(0.7)	0.3-1.5	20(2.3)	1.5-3.6	599(69.5)	66.3-72.5	
	Q3	355(39.2)	36.1-42.5	1(0.1)	0.0-0.8	20(2.2)	1.4-3.4	529(58.5)	55.2-61.6	

	Q4	611(71.3)	68.2-94.2	2(0.2)	0.1-0.9	11(1.3)	0.7-2.4	233(27.2)	24.3-30.3	
	Q5	450(90.0)	87.0-92.3	5(1.0)	0.4-2.4	5(1.0)	0.4-2.4	40(8.0)	5.9-10.7	
Zimbabwe 2010	Q1	385(45.0)	41.7-48.3	17(2.0)	1.2-3.2	7(0.8)	0.4-1.7	447(52.2)	48.9-55.6	<0.001
	Q2	396(54.0)	50.0-57.2	14(1.9)	1.1-3.2	5(0.7)	0.3-1.6	324(43.8)	40.3-47.5	
	Q3	410(61.8)	58.1-65.5	13(2.0)	1.1-3.3	5(0.8)	0.3-1.8	235(35.4)	31.9-39.2	
	Q4	622(78.7)	75.7-81.5	17(2.2)	1.3-3.4	5(0.6)	0.3-1.5	146(18.5)	15.9-31.3	
	Q5	496(89.7)	86.9-92.0	5(0.9)	0.4-2.2	1(0.2)	0.0-1.3	51(9.2)	7.1-11.9	

p: Chi-square test of heterogeneity; Q1: 20% poorest; Q5: 20% richest; CAR: Central African Republic; SBA: Skilled birth attendant

Web Figure 3a: Combination of place of delivery and type of professional, by urban/rural residence, by country.

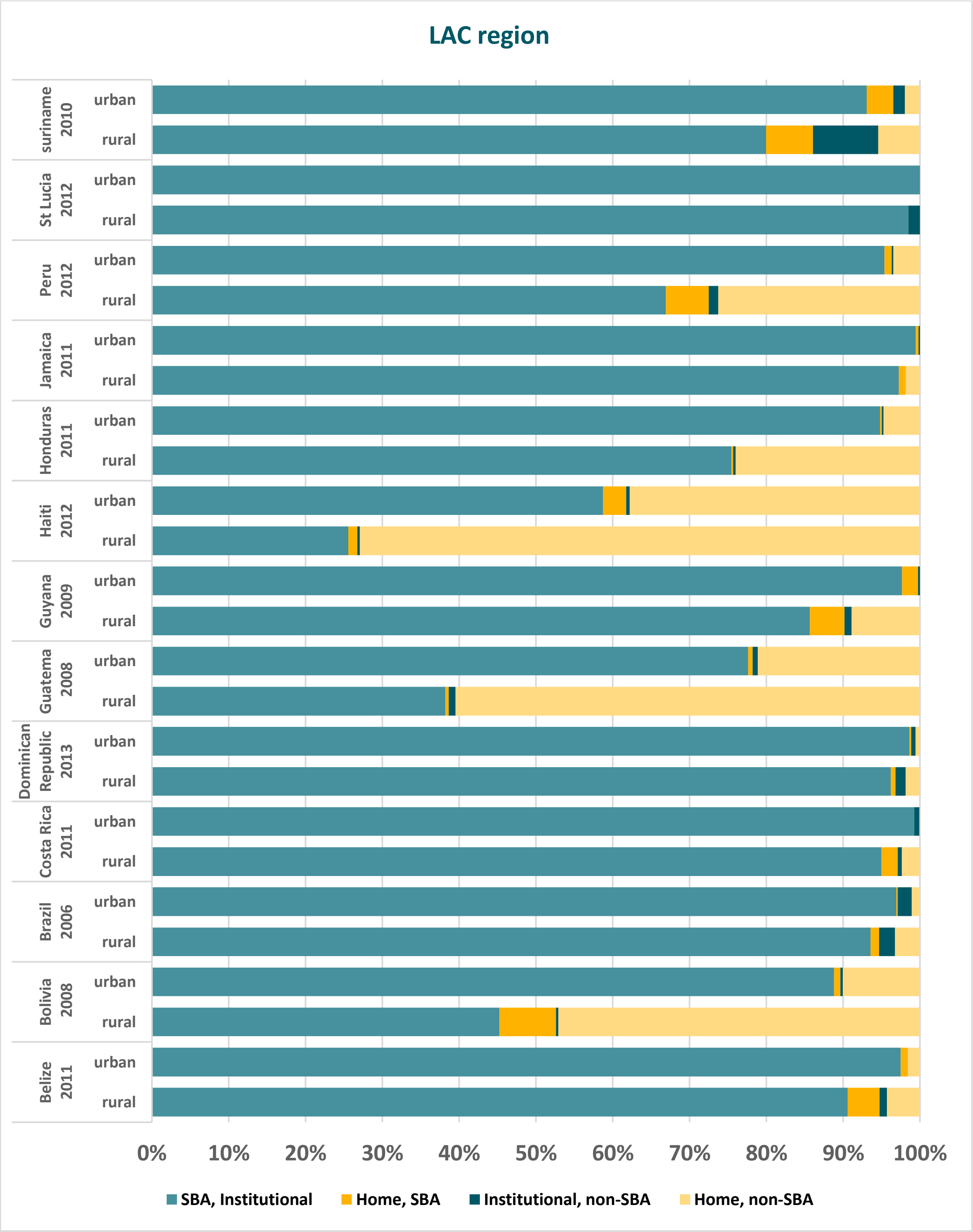

Web Figure 3b: Combination of place of delivery and type of professional, by urban/rural residence, by country.

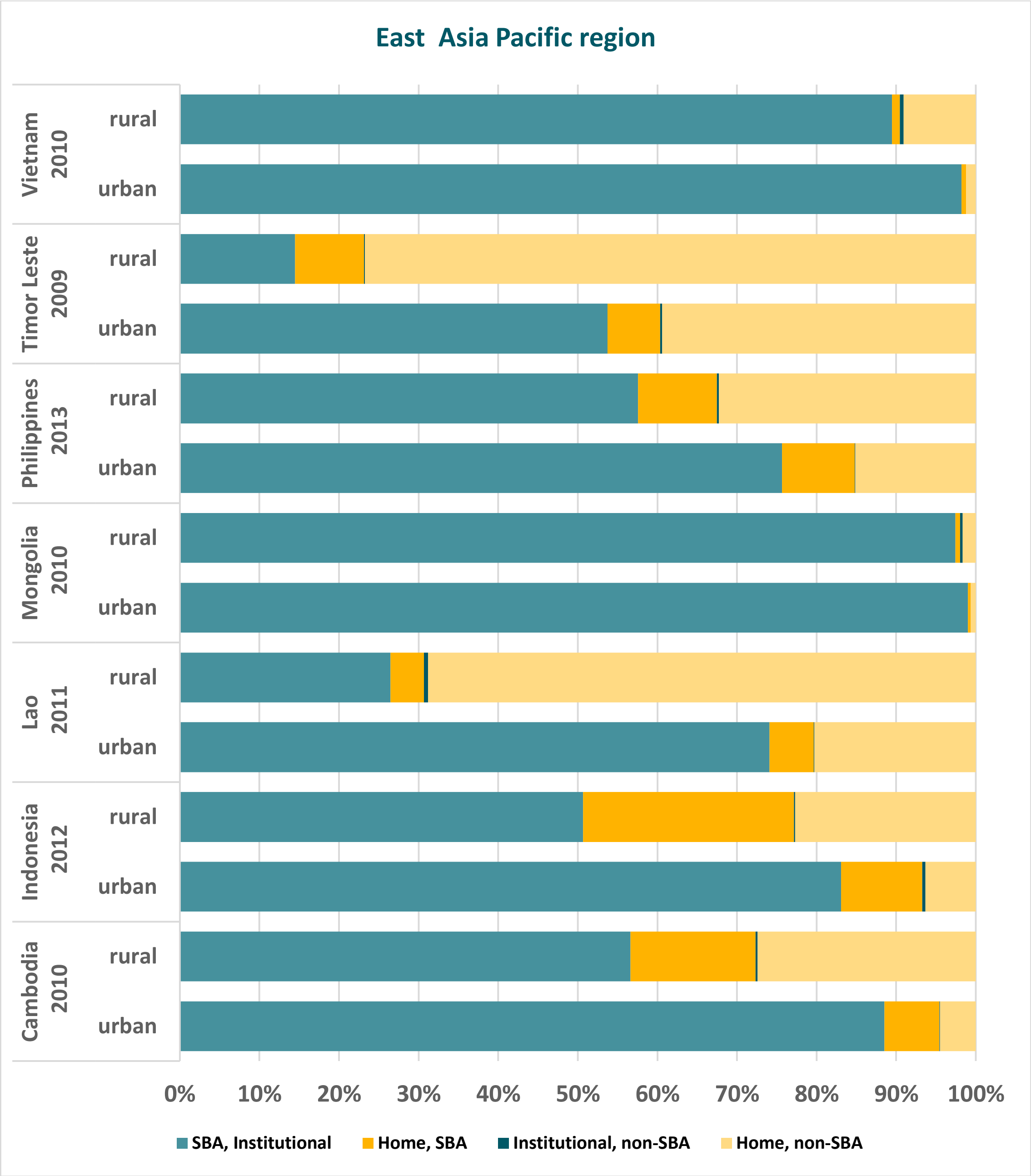

Web Figure 3c: Combination of place of delivery and type of professional, by urban/rural residence, by country.

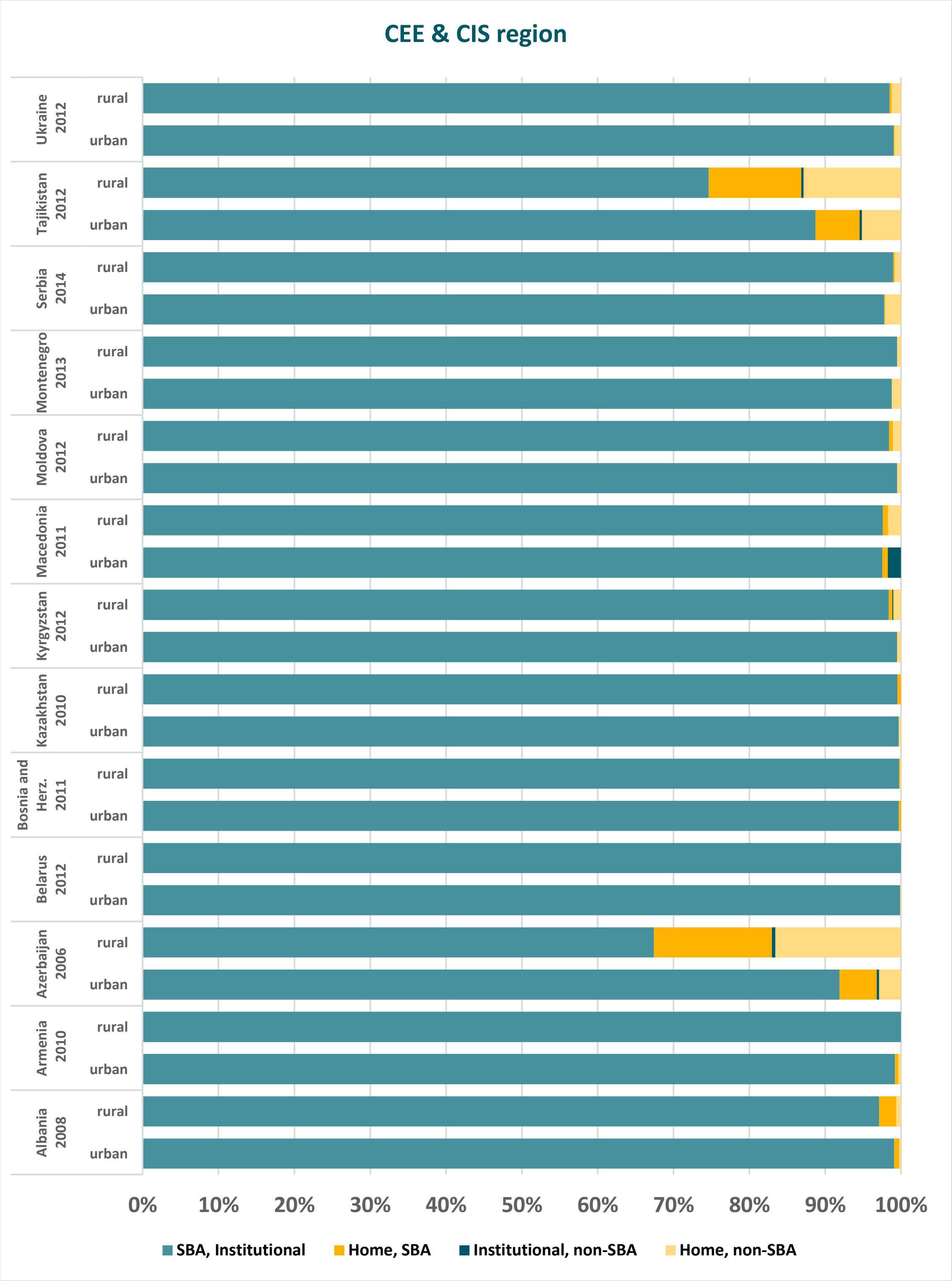

Web Figure 3d: Combination of place of delivery and type of professional, by urban/rural residence, by country.

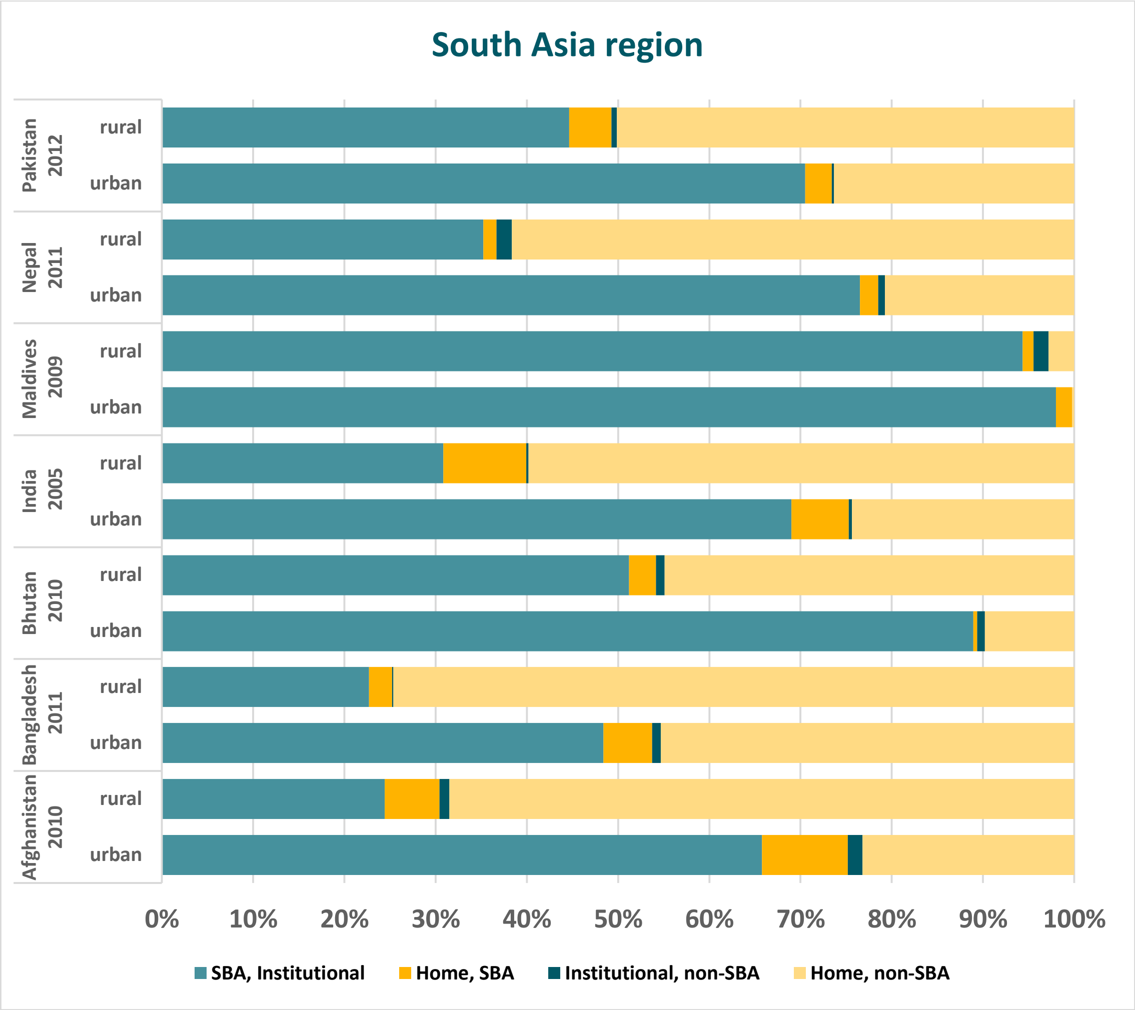

Web Figure 3e: Combination of place of delivery and type of professional, by urban/rural residence, by country.

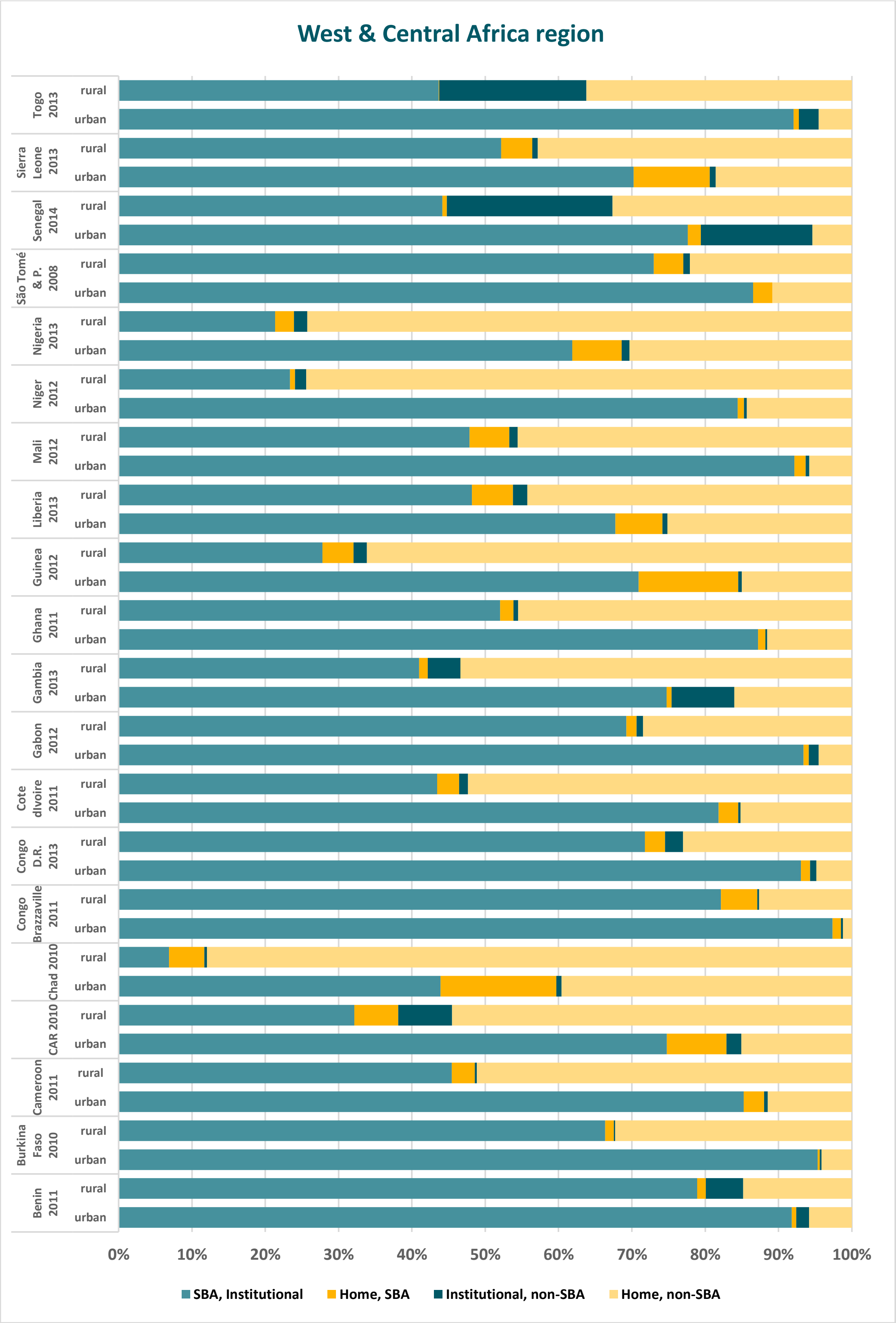

Web Figure 3f: Combination of place of delivery and type of professional, by urban/rural residence, by country.

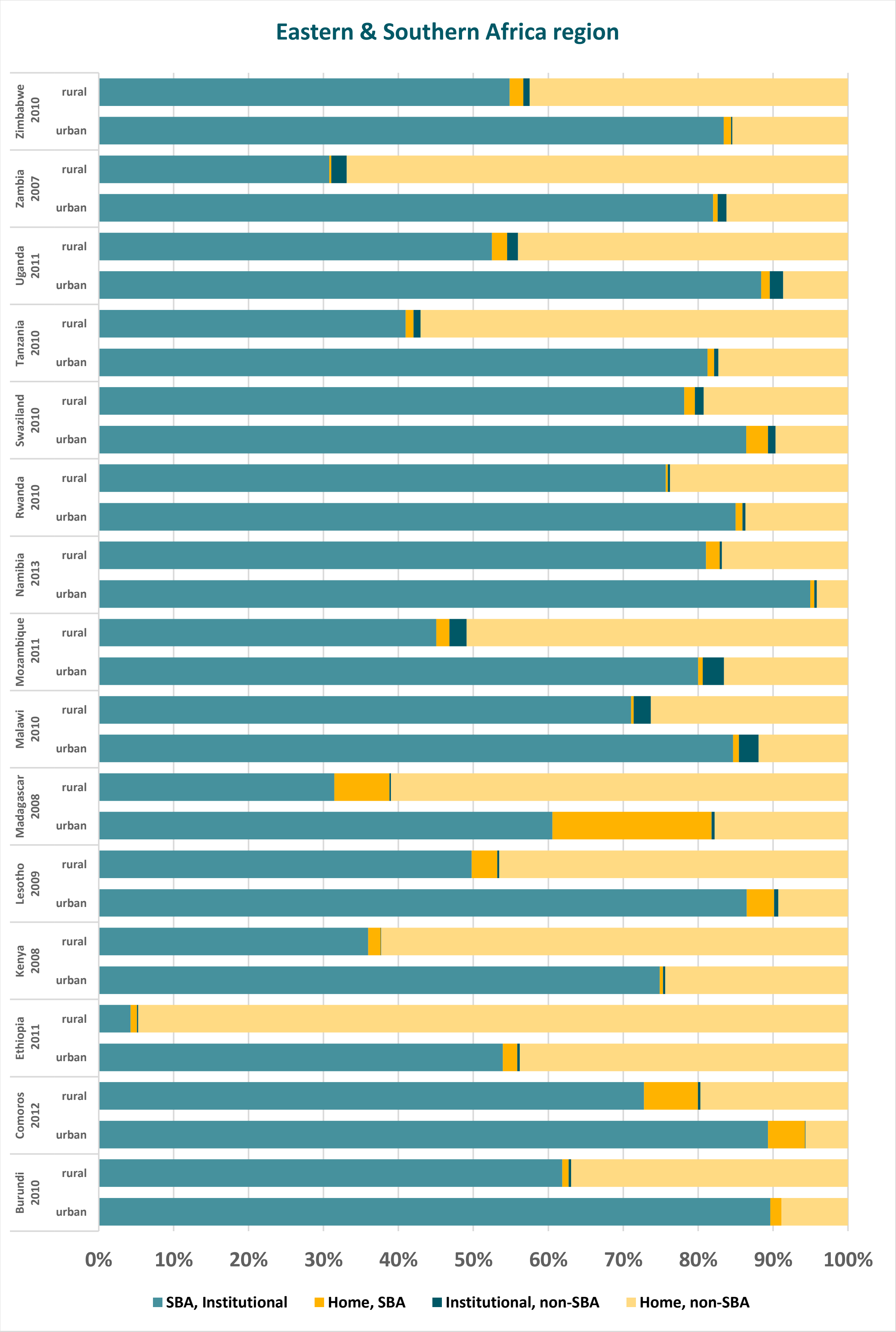

Web Figure 3g: Combination of place of delivery and type of professional, by urban/rural residence, by country.

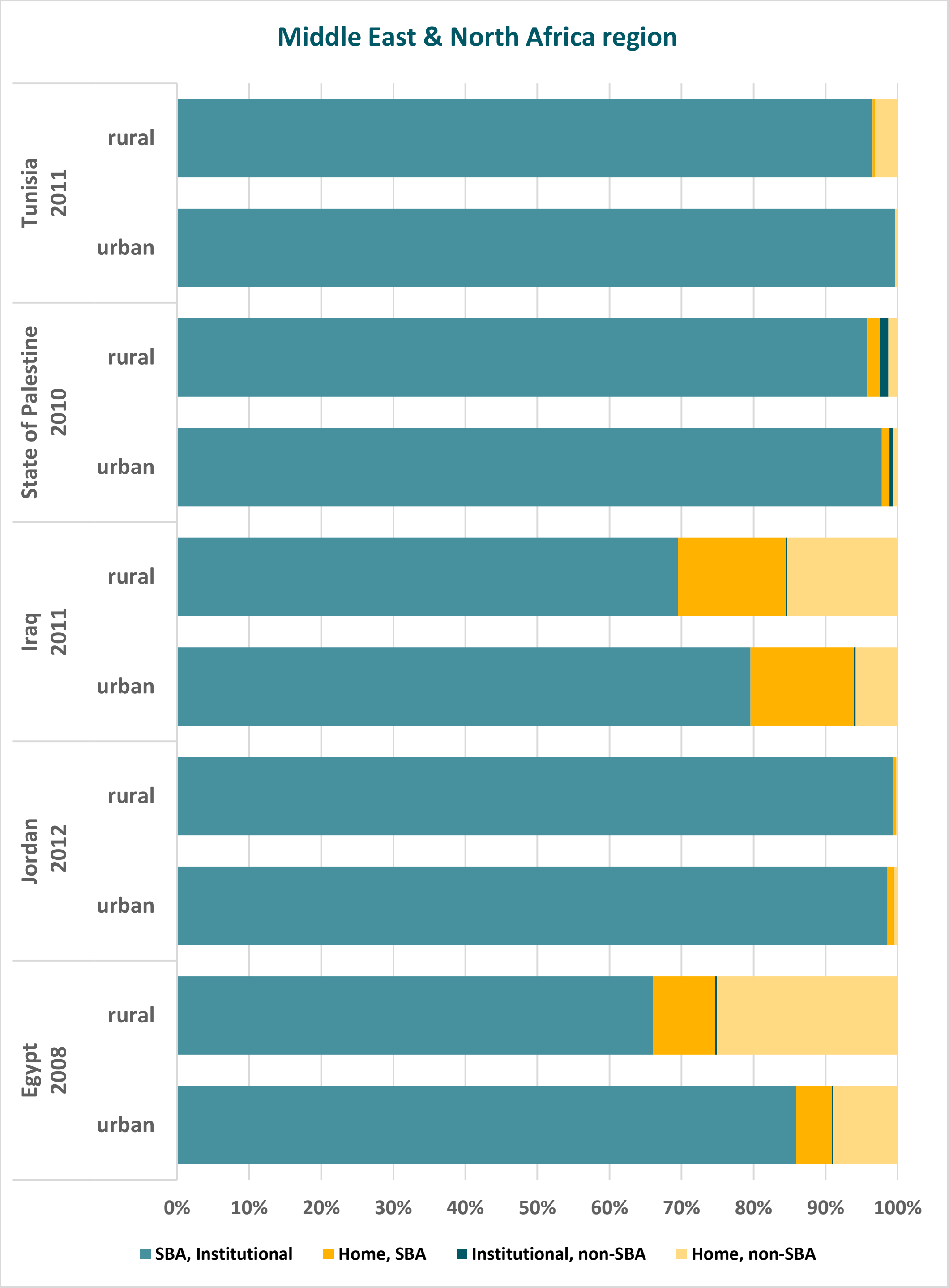

Web Figure 4a: Combination of place of delivery and type of professional, by wealth quintile, by country.

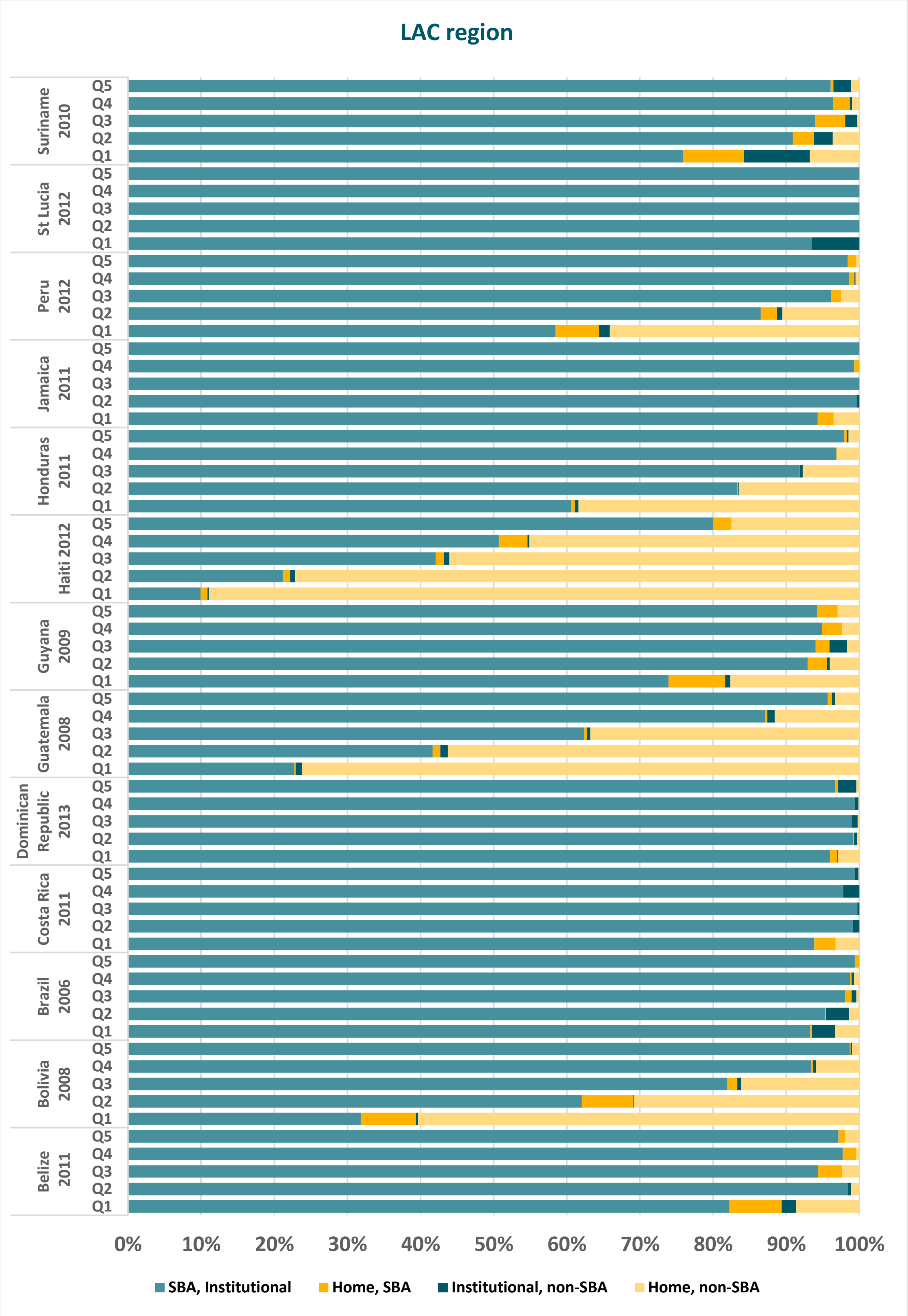

Web Figure 4b: Combination of place of delivery and type of professional, by wealth quintile, by country.

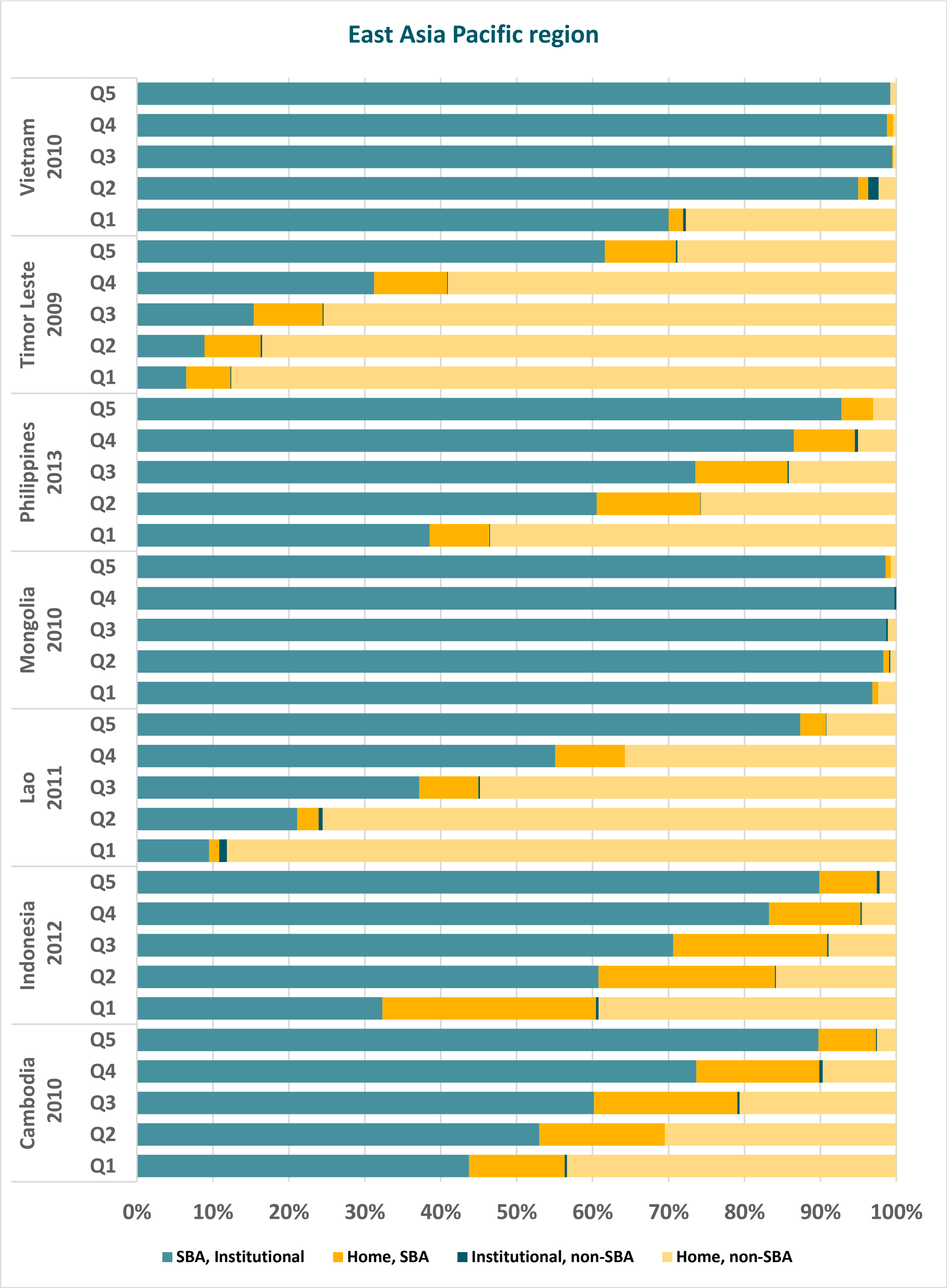

Web Figure 4c: Combination of place of delivery and type of professional, by wealth quintile, by country.

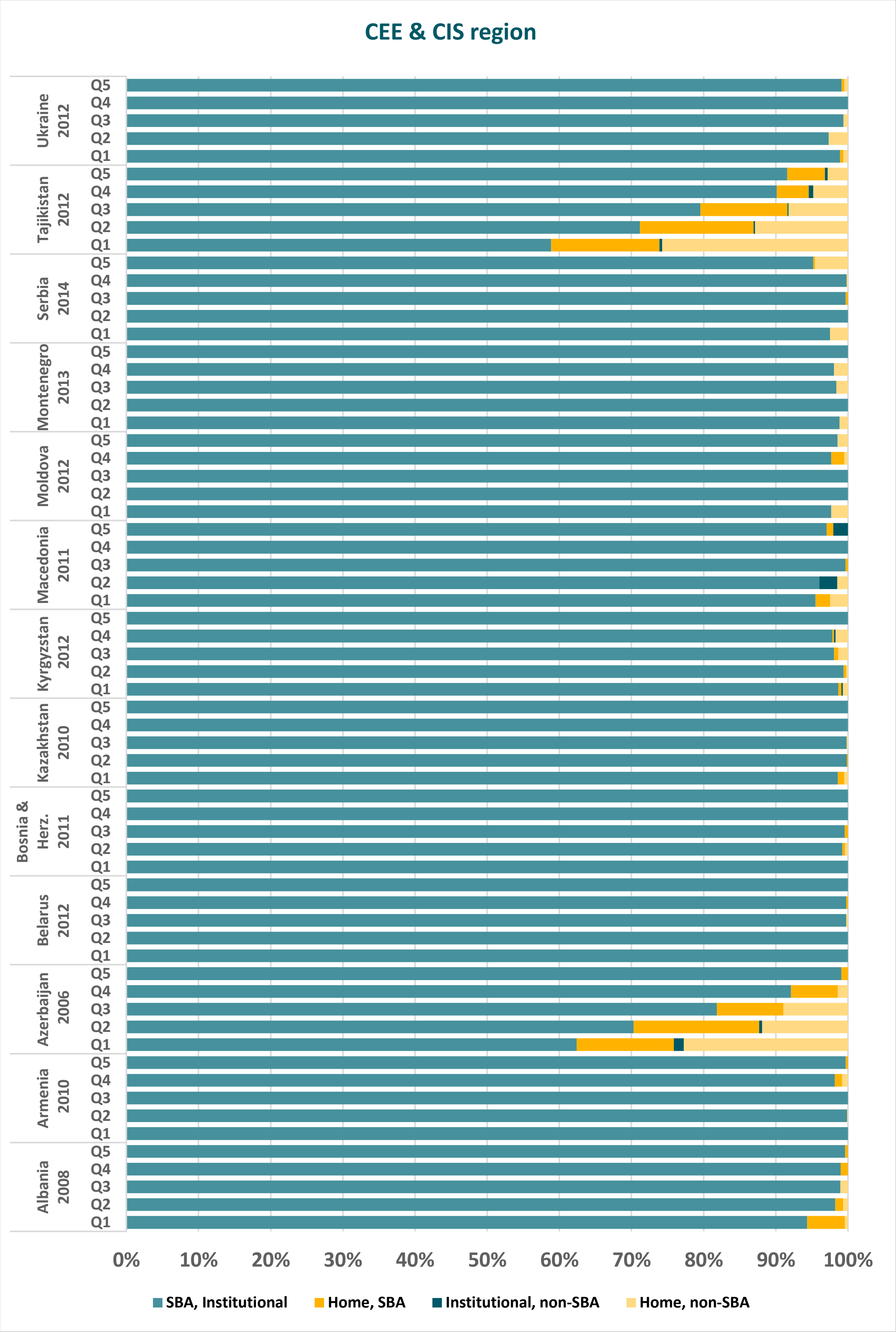

Web Figure 4d: Combination of place of delivery and type of professional, by wealth quintile, by country.

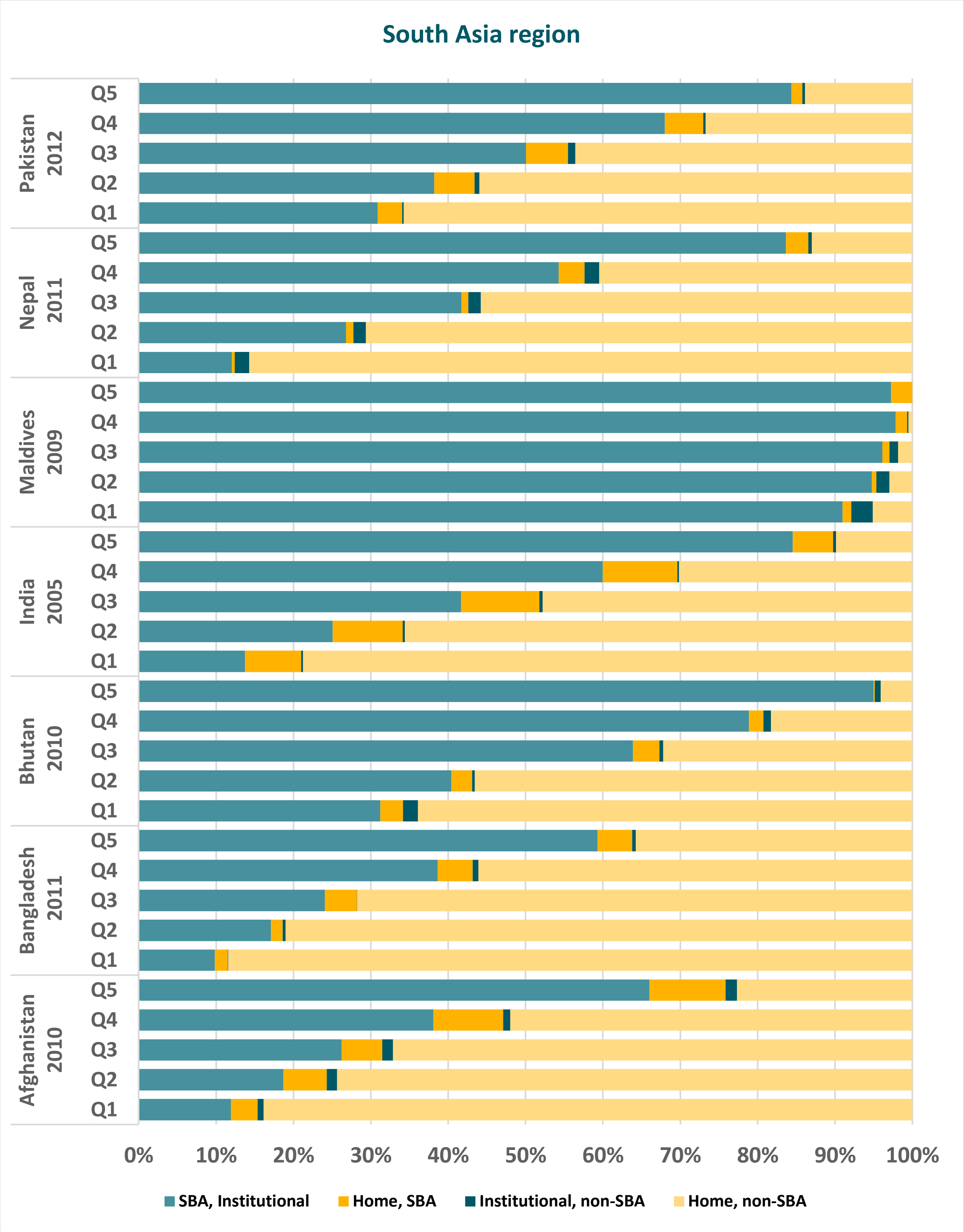

Web Figure 4e: Combination of place of delivery and type of professional, by wealth quintile, by country.

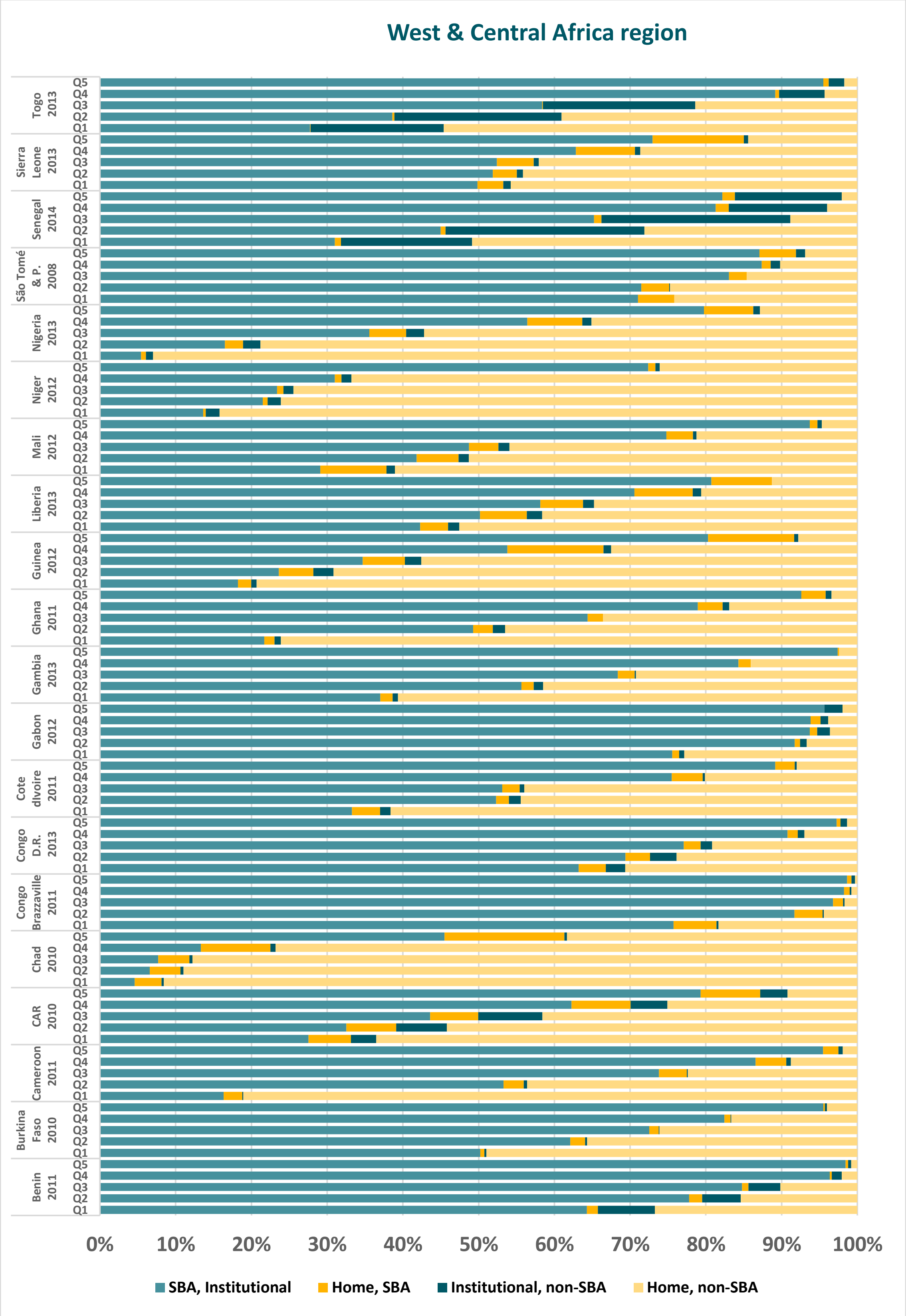

Web Figure 4f: Combination of place of delivery and type of professional, by wealth quintile, by country.

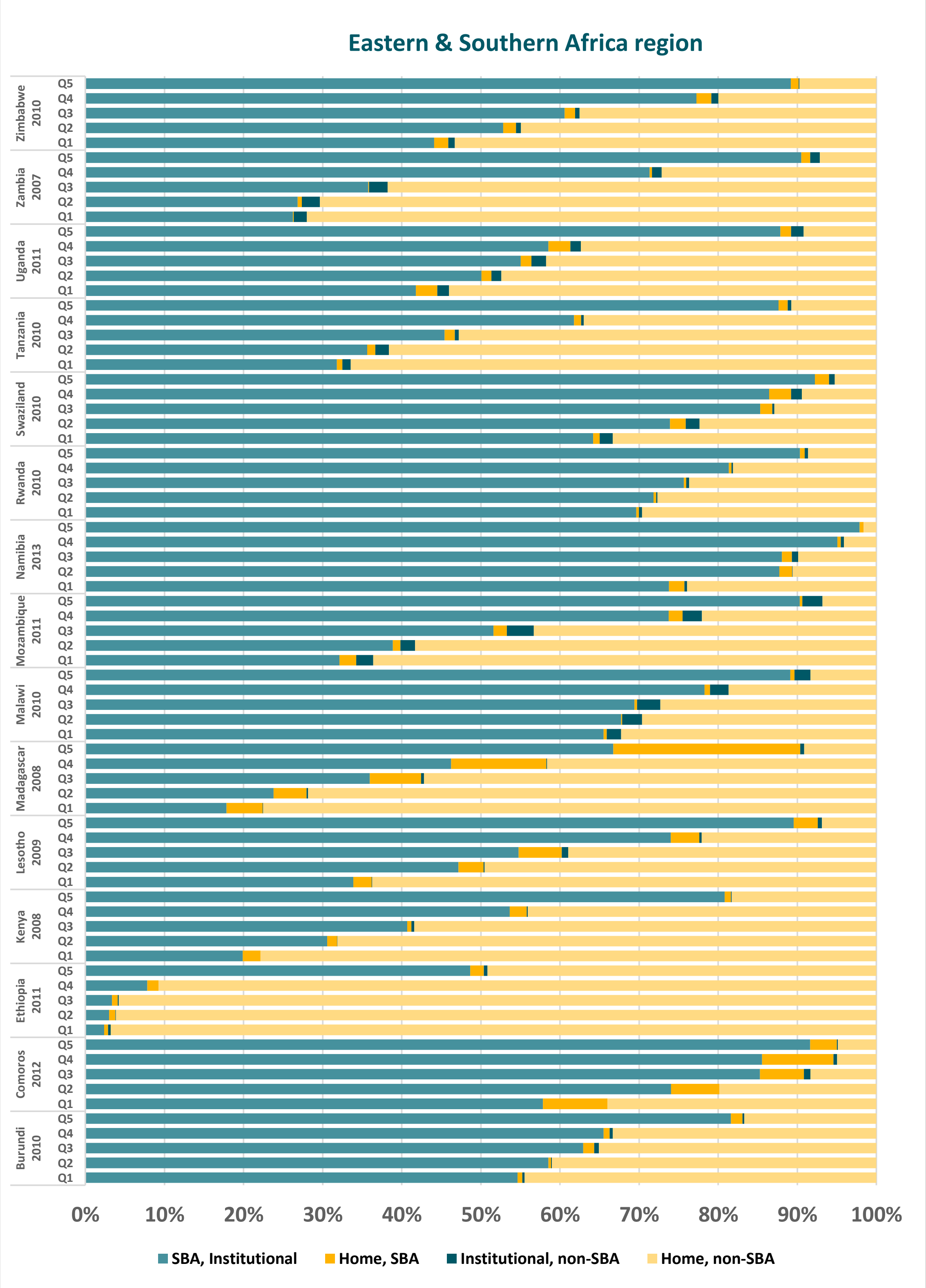

Web Figure 4g: Combination of place of delivery and type of professional, by wealth quintile, by country.

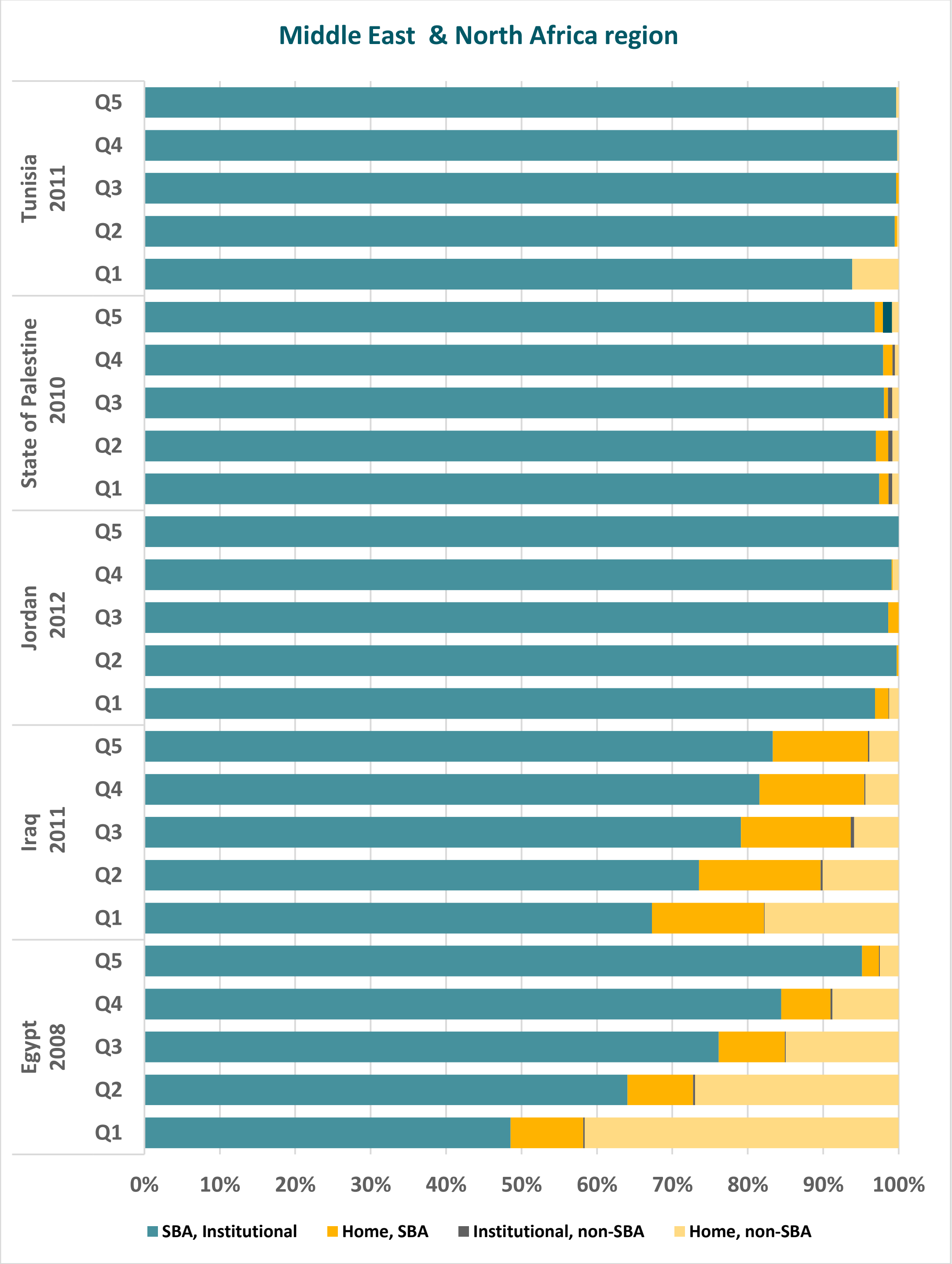

Supplement: Additional file 2: — (PDF 1141 kb) [file 12978_2016_192_MOESM2_ESM.pdf]
