# Supplementary material for: Inequalities in the coverage of place of delivery and skilled birth attendance: analyses of cross-sectional surveys in 80 low and middle-income countries
Source: Reprod Health. 2016 Jun 17;13:77. doi: 10.1186/s12978-016-0192-2 (PMC4912761; doi:10.1186/s12978-016-0192-2)
Supplement: Additional file 3: — Web appendix C: Distribution of place of delivery and type of professional in countries with at least 10 % of home SBA deliveries (Azerbaijan, Cambodia, Indonesia, Iraq, Philippines and Tajikistan) or at least 10 % of institutional deliveries by an unskilled worker (Senegal and Togo), by urban-rural residence. (PDF 217 kb) [file 12978_2016_192_MOESM3_ESM.pdf]

Web appendix C: Distribution of place of delivery and type of professional in countries with at least 10% of home SBA deliveries (Azerbaijan, Cambodia, Indonesia, Iraq, Philippines and Tajikistan) or at least 10% of institutional deliveries by an unskilled worker (Senegal and Togo), by urban-rural residence.

		Institutional, SBA			Home, SBA			Institutional, non-SBA			Home, non-SBA		
**Country**	**Place**	**N (%)**	**95% CI**	**p value**	**N (%)**	**95% CI**	**p value**	**N (%)**	**95% CI**	**p value**	**N (%)**	**95% CI**	**p value**
Azerbaijan 2006	urban	595 (91.1)	88.7-93.1	<0.001	30 (4.6)	3.2-6.5	<0.001	2 (0.3)	0.0-0.1	0.89	26 (4.0)	2.7-5.8	<0.001
	rural	544 (72.6)	69.3-75.7		103 (13.8)	11.5-16.4		2 (0.3)	0.1-1.1		100 (13.4)	11.1-16.0	
Cambodia 2010	urban	1057 (83.1)	80.9-85.1	<0.001	129 (10.1)	8.6-11.9	<0.001	5 (0.4)	0.2-1.0	0.55	81 (6.4)	5.2-7.8	<0.001
	rural	1907 (51.8)	49.8-53.0		537 (14.5)	13.4-15.7		10 (0.3)	0.1-0.5		1254 (33.8)	32.3-35.4	
Indonesia 2012	urban	3914 (79.0)	77.9-80.0	<0.001	686 (13.9)	12.9-14.8	<0.001	13 (0.3)	0.2-0.5	0.03	340 (6.9)	6.2-7.6	<0.001
	rural	2362 (39.6)	38.4-40.9		1832 (30.7)	29.6-31.9		5 (0.1)	0.0-0.2		1760 (29.5)	28.4-30.7	
Iraq 2011	urban	6097 (78.4)	77.5-79.3	<0.001	1079 (13.9)	13.1-14.7	0.93	53 (0.7)	0.5-0.9	0.001	549 (7.1)	6.5-7.6	<0.001
	rural	4164 (67.0)	65.8-68.1		859 (13.8)	13.0-14.7		18 (0.3)	0.2-0.5		1175 (18.9)	17.9-20.0	
Philippines 2013	urban	1296 (74.4)	72.3-76.4	<0.001	146 (8.4)	7.2-9.8	0.23	1 (0.1)	0.0-0.4	0.20	298 (17.1)	15.4-19.0	<0.001
	rural	1373 (54.6)	52.6-56.5		238 (9.5)	8.4-10.7		6 (0.2)	0.1-0.5		899 (35.7)	33.9-37.6	
Tajikistan 2012	urban	857 (88.7)	86.6-90.6	<0.001	58 (6.0)	4.7-7.7	<0.001	3 (0.3)	0.1-1.0	0.57	48 (5.0)	3.8-6.5	<0.001
	rural	1649 (74.0)	72.1-75.8		294 (13.2)	11.8-14.7		10 (0.5)	0.2-0.8		276 (12.4)	11.1-13.8	

Senegal 2014	urban	932 (73.9)	71.4-76.2	<0.001	22 (1.7)	1.2-2.6	0.001	201 (15.9)	14.0-18.1	<0.001	107 (8.5)	7.1-10.1	<0.001
	rural	1159 (40.3)	38.6-42.1		16 (0.6)	0.3-0.9		611 (21.3)	19.8-22.8		1087 (37.8)	36.1-39.6	
Togo 2013	urban	1130 (91.9)	90.3-93.3	<0.001	9 (0.7)	0.4-1.4	<0.001	35 (2.9)	2.1-4.0	<0.001	55 (4.5)	3.5-5.8	<0.001
	rural	1199 (39.8)	38.0-41.5		3 (0.1)	0.0-0.3		639 (21.2)	19.8-22.7		1174 (38.9)	37.3-40.7	

p: p value of Fisher's exact test; SBA: skilled birth attendant; CI: confidence interval
