# Supplementary material for: Inequalities in the coverage of place of delivery and skilled birth attendance: analyses of cross-sectional surveys in 80 low and middle-income countries
Source: Reprod Health. 2016 Jun 17;13:77. doi: 10.1186/s12978-016-0192-2 (PMC4912761; doi:10.1186/s12978-016-0192-2)
Supplement: Additional file 4: — Web appendix D: Distribution of place of delivery and type of professional in countries with at least 10 % of home SBA deliveries (Azerbaijan, Cambodia, Indonesia, Iraq, Philippines and Tajikistan) or at least 10 % of institutional deliveries by an unskilled worker (Senegal and Togo), by wealth quintile. (PDF 155 kb) [file 12978_2016_192_MOESM4_ESM.pdf]

Web appendix D: Distribution of place of delivery and type of professional in countries with at least 10% of home SBA deliveries (Azerbaijan, Cambodia, Indonesia, Iraq, Philippines and Tajikistan) or at least 10% of institutional deliveries by an unskilled worker (Senegal and Togo), by wealth quintile.

		**Institutional, SBA**			**Home, SBA**			**Institutional, non-SBA**			**Home, non-SBA**		
**Country**	**Wealth quintile**	**N (%)**	**95% CI**	**p value**	**N (%)**	**95% CI**	**p value**	**N (%)**	**95% CI**	**p value**	**N (%)**	**95% CI**	**p value**
Azerbaijan 2006	Q1 (poorest)	221 (66.6)	61.5-71.7	<0.001*	46 (13.9)	10.1-17.6	<0.001	3 (0.9)	0.1-1.9	0.03*	62 (18.7)	14.5-22.9	<0.001
	Q2	260 (74.9)	70.4-79.5		51 (14.7)	11.0-18.4		1 (0.3)	0.3-0.9		35 (10.1)	6.9-13.3	
	Q3	275 (84.6)	80.7-88.5		27 (8.3)	5.3-11.3		0 (0.0)	0.0-1.1		23 (7.1)	4.3-9.9	
	Q4	220 (94.0)	91.0-97.1		8 (3.4)	1.1-5.8		0 (0.0)	0.0-1.6		6 (2.6)	0.5-4.6	
	Q5 (richest)	163 (99.4)	98.2-1.0		1 (0.6)	0.6-1.8		0 (0.0)	0.0-2.2		0 (0.0)	0.0-2.2	
Cambodia 2010	Q1 (poorest)	495 (37.8)	35.2-40.4	<0.001	145 (11.1)	9.4-12.8	<0.001	4 (0.3)	0.0-0.6	0.40	665 (50.8)	48.1-53.5	<0.001*
	Q2	451 (46.7)	43.6-49.9		145 (15.0)	12.8-17.3		0 (0.0)	0.0-0.4		369 (38.2)	35.2-41.3	
	Q3	490 (59.2)	55.8-62.5		141 (17.0)	14.5-19.6		3 (0.4)	0.0-0.7		194 (23.4)	20.5-26.3	
	Q4	629 (73.3)	70.3-76.3		137 (16.0)	13.5-18.4		4 (0.5)	0.0-0.9		88 (10.3)	8.2-12.3	
	Q5 (richest	899 (88.1)	86.2-90.1		98 (9.6)	7.8-11.4		4 (0.4)	0.0-0.8		19 (1.9)	1.0-2.7	
Indonesia 2012	Q1 (poorest)	866 (26.4)	24.9-27.9	<0.001	960 (29.2)	27.7-30.8	<0.001	6 (0.2)	0.0-0.3	0.53	1453 (44.2)	42.5-45.9	<0.001
	Q2	1223 (55.0)	52.9-57.0		625 (28.1)	26.2-30.0		5 (0.2)	0.0-0.4		372 (16.7)	15.2-18.3	

	Q3	1326 (67.0)	65.0-69.1		478 (24.2)	22.2-26.1		2 (0.1)	0.0-0.2		172 (8.7)	7.5-9.9	
	Q4	1473 (79.8)	78.0-81.6		303 (16.4)	14.7-18.1		1 (0.1)	0.0-0.1		69 (3.7)	2.9-4.6	
	Q5 (richest	1388 (88.0)	86.4-89.6		152 (9.6)	8.2-11.1		4 (0.3)	0.0-0.5		34 (2.2)	1.4-2.9	
Iraq 2011	Q1 (poorest)	2902 (63.3)	61.9-64.6	<0.001*	652 (14.2)	13.2-15.3	0.071	11 (0.2)	0.1-0.4	0.001	1022 (22.3)	21.1-23.5	<0.001
	Q2	2411 (73.9)	72.4-75.4		473 (14.5)	13.3-15.7		26 (0.8)	0.5-1.2		353 (10.8)	9.8-11.9	
	Q3	2063 (78.1)	76.5-79.7		378 (14.3)	13.0-15.7		21 (0.8)	0.5-1.2		179 (6.8)	5.9-7.8	
	Q4	1641 (81.2)	79.5-82.9		258 (12.8)	11.4-14.3		8 (0.4)	0.2-0.8		113 (5.6)	4.7-6.7	
	Q5 (richest)	1244 (83.9)	81.9-85.7		177 (11.9)	10.4-13.7		5 (0.3)	0.1-0.8		57 (3.8)	3.0-5.0	
Philippines 2013	Q1 (poorest)	481 (35.9)	33.3-38.4	<0.001	102 (7.6)	6.2-9.0	<0.001	1 (0.1)	0.1-0.2	0.269	757 (56.5)	53.8-59.1	<0.001
	Q2	550 (58.8)	55.7-62.0		119 (12.7)	10.6-14.9		1 (0.1)	0.1-0.3		265 (28.3)	25.5-31.2	
	Q3	612 (73.3)	70.3-76.3		96 (11.5)	9.3-13.7		2 (0.2)	0.1-0.6		125 (15.0)	12.5-17.4	
	Q4	583 (86.9)	84.3-89.4		49 (7.3)	5.3-9.3		3 (0.5)	0.1-1.0		36 (5.4)	3.7-7.1	
	Q5 (richest)	443 (93.3)	91.0-95.5		18 (3.8)	2.1-5.5		0 (0.0)	0.0-0.8		14 (3.0)	1.4-4.5	
Tajikistan 2012	Q1 (poorest)	325 (58.9)	54.8-63.0	<0.001	91 (16.5)	13.4-19.6	<0.001	3 (0.5)	0.1-1.2	0.791	133 (24.1)	20.5-27.7	<0.001
	Q2	424 (69.2)	65.5-72.8		104 (17.0)	14.0-20.0		2 (0.3)	0.2-0.8		83 (13.5)	10.8-16.3	
	Q3	503 (78.7)	75.5-82.0		84 (13.2)	10.5-15.8		2 (0.3)	0.1-1.0		50 (7.8)	5.7-9.9	

	Q4	557 (88.6)	86.1-91.0		33 (5.3)	3.5-7.0		4 (0.6)	0.0-1.3		35 (5.6)	3.8-7.4	
	Q5 (richest	697 (91.5)	89.5-93.5		40 (5.3)	3.7-6.8		2 (0.3)	0.1-0.6		23 (3.0)	1.8-4.2	
Senegal 2014	Q1 (poorest)	421 (30.1)	27.7-32.5	<0.001	12 (0.9)	0.4-1.3	0.16	239 (17.1)	15.1-19.1	<0.001	727 (52.0)	49.3-54.6	<0.001
	Q2	497 (44.8)	41.8-47.7		7 (0.6)	0.2-1.1		265 (23.9)	21.4-26.4		341 (30.7)	28.0-33.4	
	Q3	533 (64.7)	61.4-68.0		7 (0.9)	0.2-1.5		194 (23.5)	20.6-26.4		90 (10.9)	8.8-13.1	
	Q4	377 (79.2)	75.5-82.9		5 (1.1)	0.1-2.0		66 (13.9)	10.8-17.0		28 (5.9)	3.8-8.0	
	Q5 (richest	263 (80.7)	76.4-85.0		7 (2.2)	0.6-3.7		48 (14.7)	10.9-18.6		8 (2.5)	0.8-4.1	
Togo 2013	Q1 (poorest)	341 (27.5)	25.0-30.0	<0.001	1 (0.1)	0.1-0.2	0.063	217 (17.5)	15.4-19.6	<0.001	681 (54.9)	52.1-57.7	<0.001
	Q2	332 (38.3)	35.1-41.5		2 (0.2)	0.1-0.6		198 (22.9)	20.2-25.8		334 (38.6)	35.4-41.9	
	Q3	457 (55.2)	51.7-58.5		1 (0.1)	0.1-0.4		198 (23.9)	21.1-26.9		172 (20.8)	18.1-23.6	
	Q4	590 (88.2)	85.7-90.6		3 (0.5)	0.1-1.0		46 (6.9)	5.0-8.8		30 (4.5)	2.9-6.1	
	Q5 (richest)	609 (95.1)	93.3-96.7		5 (0.8)	0.1-1.5		15 (2.3)	1.2-3.5		12 (1.9)	0.8-2.9	

* p value for chi-square test for linear trend; SBA: skilled birth attendant
