# Supplementary material for: Inequalities in the coverage of place of delivery and skilled birth attendance: analyses of cross-sectional surveys in 80 low and middle-income countries
Source: Reprod Health. 2016 Jun 17;13:77. doi: 10.1186/s12978-016-0192-2 (PMC4912761; doi:10.1186/s12978-016-0192-2)
Supplement: Additional file 5: — Web appendix B: ISO codes. (PDF 196 kb) [file 12978_2016_192_MOESM5_ESM.pdf]

Appendix B: ISO codes

ISO codes	Country	ISO codes	Country	ISO codes	Country	ISO codes	Country
AFG	Afghanistan	COD	Congo Democratic Republic	KEN	Kenya	PHL	Philippines
ALB	Albania	CRI	Costa Rica	KGZ	Kyrgyzstan	RWA	Rwanda
ARM	Armenia	CIV	Cote dIvoire	LAO	Lao	STP	São Tome & Principe
AZE	Azerbaijan	DOM	Dominican Republic	LSO	Lesotho	SEN	Senegal
BGD	Bangladesh	EGY	Egypt	LBR	Liberia	SRB	Serbia
BLR	Belarus	ETH	Ethiopia	MKD	Macedonia	SLE	Sierra Leone
BLZ	Belize	GAB	Gabon	MDG	Madagascar	LCA	St Lucia
BEN	Benin	GMB	Gambia	MWI	Malawi	PSE	State of Palestine
BTN	Bhutan	GHA	Ghana	MDV	Maldives	SUR	Suriname
BOL	Bolivia	GTM	Guatemala	MLI	Mali	SWZ	Swaziland
BIH	Bosnia and Herzegovina	GIN	Guinea	MDA	Moldova	TJK	Tajikistan
BRA	Brazil	GUY	Guyana	MNG	Mongolia	TZA	Tanzania
BFA	Burkina Faso	HTI	Haiti	MNE	Montenegro	TLS	Timor Leste
BDI	Burundi	HND	Honduras	MOZ	Mozambique	TGO	Togo
KHM	Cambodia	IND	India	NAM	Namibia	TUN	Tunisia
CMR	Cameroon	IDN	Indonesia	NEP	Nepal	UGA	Uganda
CAF	CAR	IRQ	Iraq	NER	Niger	UKR	Ukraine
TCD	Chad	JAM	Jamaica	NGA	Nigeria	VNM	Vietnam
COM	Comoros	JOR	Jordan	PAK	Pakistan	ZMB	Zambia
COG	Congo Brazzaville	KAZ	Kazakhstan	PER	Peru	ZWE	Zimbabwe
